# Probing the Inhibitor versus Chaperone Properties of sp^2^-Iminosugars towards Human β-Glucocerebrosidase: A Picomolar Chaperone for Gaucher Disease

**DOI:** 10.3390/molecules23040927

**Published:** 2018-04-17

**Authors:** Teresa Mena-Barragán, M. Isabel García-Moreno, Alen Sevšek, Tetsuya Okazaki, Eiji Nanba, Katsumi Higaki, Nathaniel I. Martin, Roland J. Pieters, José M. García Fernández, Carmen Ortiz Mellet

**Affiliations:** 1 Department of Organic Chemistry, Faculty of Chemistry, University of Sevilla, C/Profesor García González 1, 41011 Sevilla, Spain; tmena@us.es (T.M.-B.); isagar@us.es (M.I.G.-M.); 2 Department of Chemical Biology & Drug Discovery, Utrecht Institute for Pharmaceutical Sciences, Utrecht University, Universiteitsweg 99, 3584 CG Utrecht, The Netherlands; Sevsek@ke-instruments.com; 3 Division of Child Neurology, Department of Brain and Neurosciences, Faculty of Medicine, Tottori University, Yonago 680-8550, Japan; t-okazaki@med.tottori-u.ac.jp; 4 Division of Functional Genomics, Research Center for Bioscience and Technology, Tottori University, 86 Nishi-cho, Yonago 683-8503, Japan; enanba@med.tottori-u.ac.jp; 5 Instituto de Investigaciones Químicas (IIQ), CSIC—University of Sevilla, Avda. Americo Vespucio 49, 41092 Sevilla, Spain

**Keywords:** sp^2^-Iminosugars, deoxynojirimycin, glycosidase inhibitors, glucocerebrosidase, chaperones, Gaucher disease

## Abstract

A series of sp^2^-iminosugar glycomimetics differing in the reducing or nonreducing character, the configurational pattern (d-*gluco* or l-*ido*), the architecture of the glycone skeleton, and the nature of the nonglycone substituent has been synthesized and assayed for their inhibition properties towards commercial glycosidases. On the basis of their affinity and selectivity towards GH1 β-glucosidases, reducing and nonreducing bicyclic derivatives having a hydroxylation profile of structural complementarity with d-glucose and incorporating an *N′*-octyl-isourea or -isothiourea segment were selected for further evaluation of their inhibitory/chaperoning potential against human glucocerebrosidase (GCase). The 1-deoxynojirimycin (DNJ)-related nonreducing conjugates behaved as stronger GCase inhibitors than the reducing counterparts and exhibited potent chaperoning capabilities in Gaucher fibroblasts hosting the neuronopathic G188S/G183W mutation, the isothiourea derivative being indeed one of the most efficient chaperone candidates reported up to date (70% activity enhancement at 20 pM). At their optimal concentration, the four selected compounds promoted mutant GCase activity enhancements over 3-fold; yet, the inhibitor/chaperoning balance became unfavorable at much lower concentration for nonreducing as compared to reducing derivatives.

## 1. Introduction

**Figure 1:**
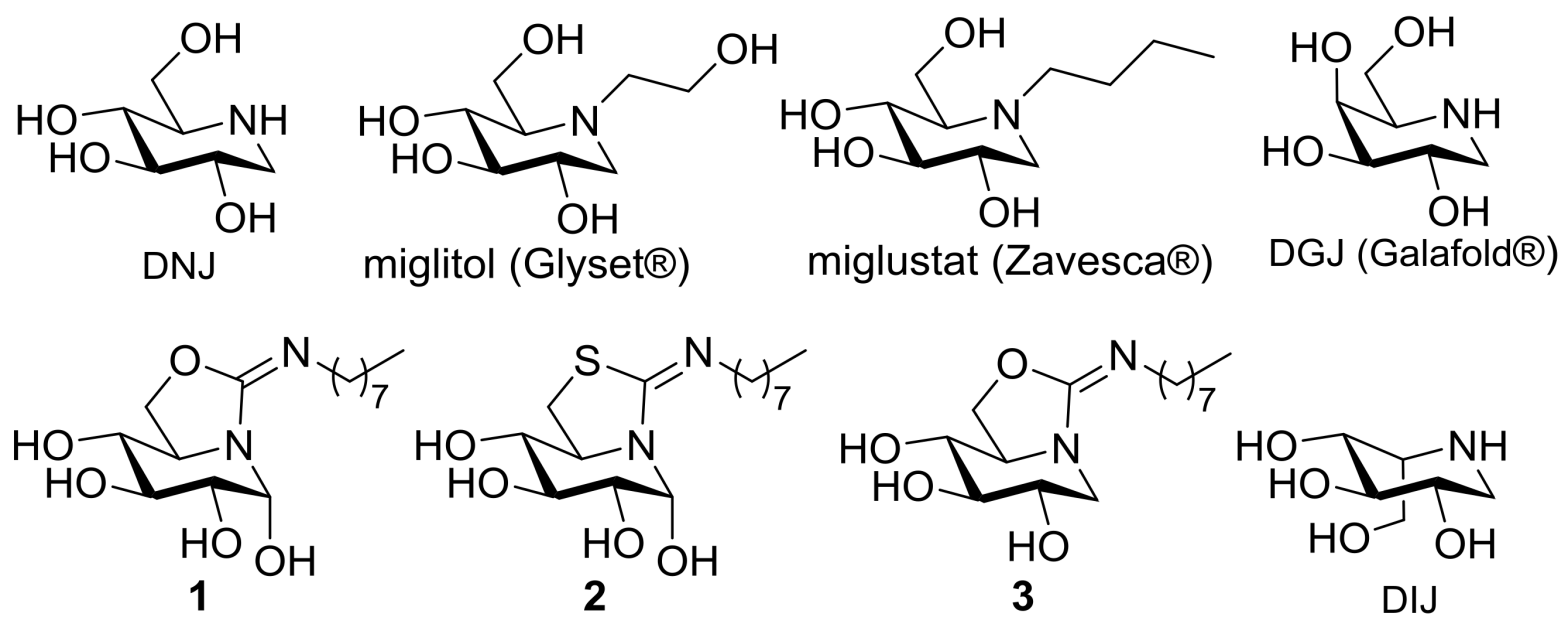
Structures of the iminosugars 1-deoxynojirimycin (DNJ), 1-deoxygalactonojirimycin (DGJ) and 1-deoxy-l-idonojirimycin (DIJ), the DNJ-related drugs miglitol and miglustat, and the sp^2^-iminosugars **1**–**3**.

Structural analogues of carbohydrates, generically termed glycomimetics, with the ability to interfere with sugar-processing enzymes (glycosidases, glycosyltransferases) have proven extremely useful for the investigation of mechanistic aspects pertaining to enzymatic glycoside hydrolysis or formation and the biochemical routes in which oligosaccharides and/or glycoconjugates are involved. Considering the utmost importance of such processes in cell biology, it is not surprising that the dysfunction of glycosidases or glycosyltransferases often results in life-threatening diseases in humans, ranging from diabetes to neurological diseases or cancer development and metastasis. Consequently, modulation of their activity by using glycomimetics bears strong potential for the development of therapies against a broad range of conditions [1]. Polyhydroxylated alkaloids of the iminosugar family are by far the most intensely studied compounds in this regard, with some products being currently marketed as drugs [2,3,4]. Thus, the *N*-substituted 1-deoxynojirimycin (DNJ) derivative miglitol (Glyset^®^, Pfizer, New York, NY, USA), an inhibitor of the intestinal α-glucosidases, is used for glycemic control in the management of type 2 diabetes mellitus [5], whereas *N*-butyl-DNJ (miglustat; Zavesca^®^, Actelion Pharmaceuticals Ltd., Allschwil, Switzerland), which inhibits glucosylceramide synthase, is indicated in the treatment of Gaucher disease, a lysosomal storage disorder (LSD) resulting from the malfunctioning of the acid β-glucosidase (β-glucocerebrosidase, GCase) [6]. Very recently, the DNJ epimer 1-deoxygalactonojirimycin (DGJ), as the corresponding hydrochloride salt (migalastat; Galafold^®^, Amicus Therapeutics, Cranbury, NJ, USA), was approved by the European Medicine Agency (EMA) for the treatment of some forms of Fabry disease, another LSD (Figure 1) [7,8]. In this case, mutations in the gene encoding for acid α-galactosidase leads to a misfolded mutant protein, ensuing endoplasmic reticulum associated degradation (ERAD), and the accumulation of its putative substrate in the lysosomes. DGJ behaves as an α-galactosidase inhibitor in vitro; yet, it acts as an effector of the mutant enzyme in vivo by promoting its correct folding and trafficking after binding at the active site [9,10,11]. Once at the lysosome, the excess of substrate displaces the glycomimetic and the hydrolytic activity is restored, a rescuing mechanism known as pharmacological chaperone therapy [12,13,14,15]. The pharmacological chaperone concept constitutes a new therapeutic paradigm potentially applicable to the whole range of protein misfolding diseases, which, in addition to the LSDs, include Alzheimer’s, Parkinson’s, Huntington’s, or amyotrophic lateral sclerosis diseases, to cite just a few [16,17,18].

Two important prerequisites for a glycomimetic becoming a pharmacological chaperone candidate for a given LSD are high selectivity towards the lysosomal enzyme target and high endoplasmic reticulum (pH 7) versus lysosome (pH 4.5) binding affinity ratio [19,20]. The conversion of the amine-type endocyclic nitrogen of iminosugars into a pseudoamide-type functionality, with high sp^2^-hybridation character (sp^2^-iminosugars) [21,22], has demonstrated to be a very promising strategy to improve both parameters, with several sp^2^-iminosugar representatives under investigational or preclinical development for the LSDs Gaucher [23,24,25,26,27], Fabry [28], G_M1_-gangliosidosis [29,30], and Tay-Sachs diseases [31]. The glycosidase inhibitory and chaperoning abilities of sp^2^-iminosugars have been found to be strongly dependent on the mono- [32,33,34,35,36] or bicyclic structure of the sugar glycone-like skeleton [37,38,39,40,41,42,43,44], the configurational pattern [45,46,47,48], and the nature of nonglycone-type substituents [49,50,51]. Differently from classical iminosugars, for which the lability of aminal functionalities prevents the installation of anomeric substituents (except in the case of pseudo-*C*-glycosides) [52,53,54,55], sp^2^-iminosugars can bear *O*-, *S*-, *N*-, or *C*-anomeric aglycone groups while keeping full chemical and configurational stability [56,57,58,59,60,61,62,63,64,65], even when multivalently presented [66,67,68,69]. The possibility of accessing reducing analogues with a precise α-like axial orientation of the anomeric hydroxyl is quite unique [70,71,72]. Interestingly, α-reducing derivatives such as **1** and **2** (Figure 1) were found to behave as very selective inhibitors of the commercial β-glucosidases from almonds, bovine liver, and *Thermotoga maritima* in a preliminary screening against a panel of commercial glycosidases, and as very efficient chaperones for a series of mutant GCase forms associated to Gaucher disease [23,73,74]. All these β-glucosidases belong to the glycosyl hydrolase family GH1 in the CAZy classification [75], meaning that they share considerable analogy in their active site architecture. X-ray structural studies evidenced that in the corresponding enzyme:glycomimetic complexes the anomeric configuration switch into β [76] or, if remaining α, the OH-group adopts an equatorial disposition to avoid steric clashes [77]. No productive interactions with amino acid residues of the proteins were identified at this region, however, which raises the question of whether or not the presence of the anomeric hydroxyl has a beneficial effect regarding the chaperoning behavior. As a matter of fact, the DNJ-related derivative **3** was recently found to be also a very potent GCase competitive inhibitor [78]. It should be also noted that GH1 β-glucosidases are particularly tolerant to configurational modifications in their binding partners: in addition to substrate/inhibitors with a hydroxylation profile of structural complementarity to d-glucose, they can accept ligands with d-*galacto* and l-*ido* configurational profiles. Iminosugars and sp^2^-iminosugars with the latter configuration, for instance 1-deoxy-l-idonojirimycin (DIJ) derivatives, have been found particularly interesting for chaperone therapy purposes given their high selectivity towards GCase, among other enzymes involved in glucosylceramide metabolisms (i.e., cytosolic β-glucosidase and glucosylceramide synthase) [79,80].

**Figure 2:**
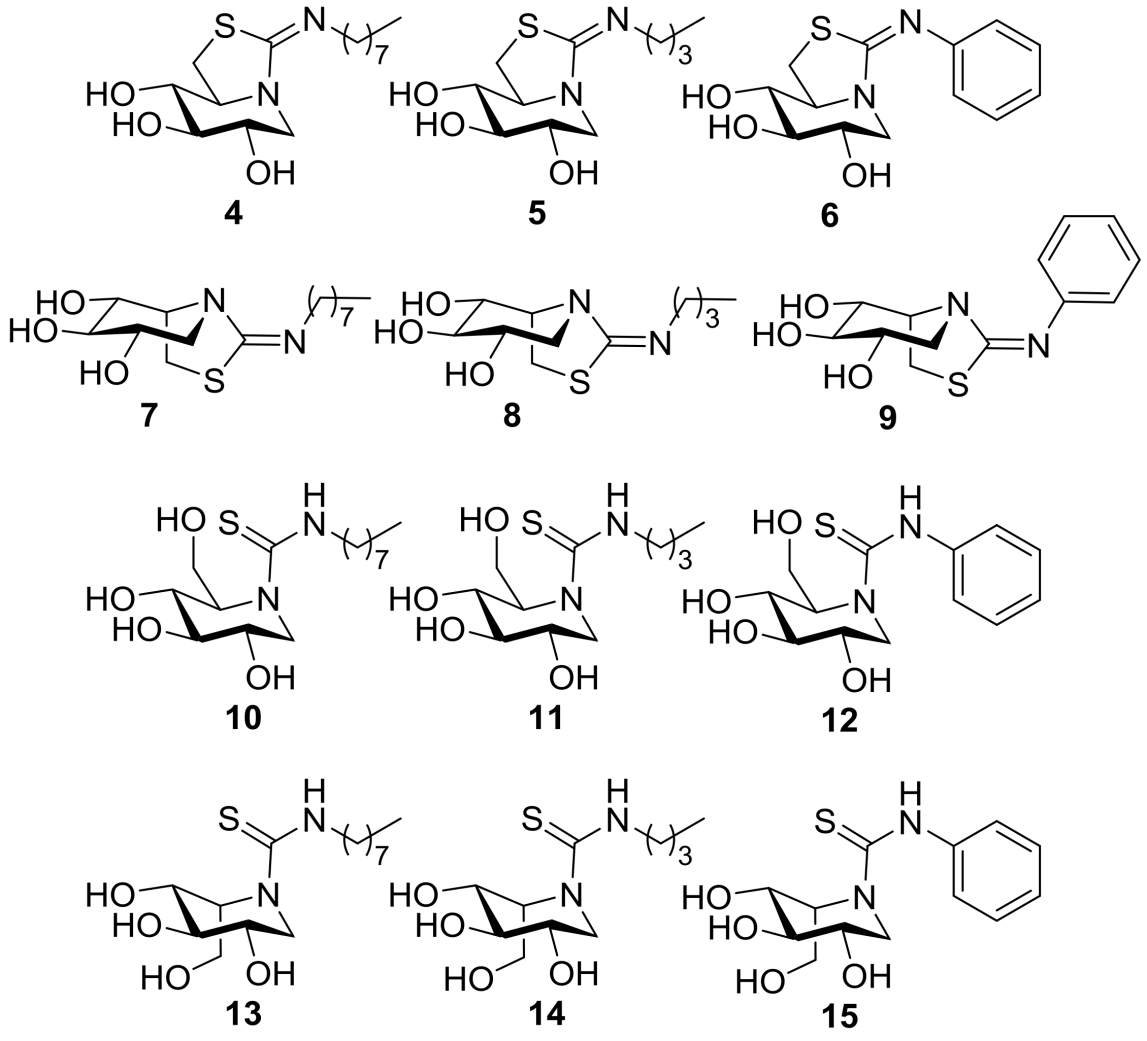
Structures of the new sp^2^-iminosugars prepared in this work.

In this work, we wanted to assess the effect of structural modifications in sp^2^-iminosugars in their β-glucosidase inhibitory properties and in their chaperoning capabilities towards Gaucher disease-causative mutant GCase forms. More precisely, we have considered the series of compounds **1**–**15** (Figure 2) to evaluate the impact of (a) the reducing or nonreducing character (**1** and **2** vs. **3** and **4**), (b) the bicyclic or monocyclic nature (**4**–**9** vs. **10**–**15**), (c) the configuration at the position equivalent to C-5 in monosaccharides (DNJ or DIJ derivatives; **4**–**6** and **10**–**12** vs. **7**–**9** and **13**–**15**), and (d) the nature of nonglycone-type substituents in the inhibitory/chaperoning properties (octyl, butyl, or phenyl).

## 2. Results

### 2.1. Synthesis

**Scheme 1:**
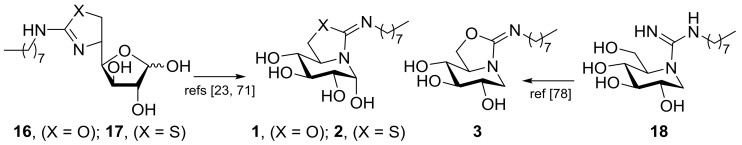
Key synthetic steps for the synthesis of compounds **1**–**3**.

The reducing bicyclic derivatives **1** and **2**, differing in the presence of an oxygen or a sulfur atom in the five-membered ring, respectively, were synthesized through a previously reported reaction sequence that implies, as the key step, the spontaneous rearrangement of 5*N*,6*O*- or 6*S*-(cyclic isourea or isothiourea)-d-glucofuranose precursors (**16** or **17**, respectively) by intramolecular nucleophilic attack of the endocyclic nitrogen atom to the masked aldehyde group of the monosaccharide [23,71]. The resulting hemiaminal carbon adopts exclusively the α-configuration dictated by the generalized anomeric effect in water solution as a consequence of a very efficient overlapping between the p orbital hosting the lone electron pair of the sp^2^-hybridized nitrogen atom and the σ* antibonding orbital of the pseudoanomeric C–O bond. The nonreducing 5*N*,6*O*-*N′*-octyliminomethylidene DNJ derivative **3** is the 1-deoxy nonreducing homologue of **1**. It was accessed via the *N*′-octylguanidine DNJ intermediate **18** by spontaneous conversion, implying addition of the primary OH-6 oxygen to the guanidine carbon and elimination of ammonia as reported (Scheme 1) [78].

**Scheme 2:**
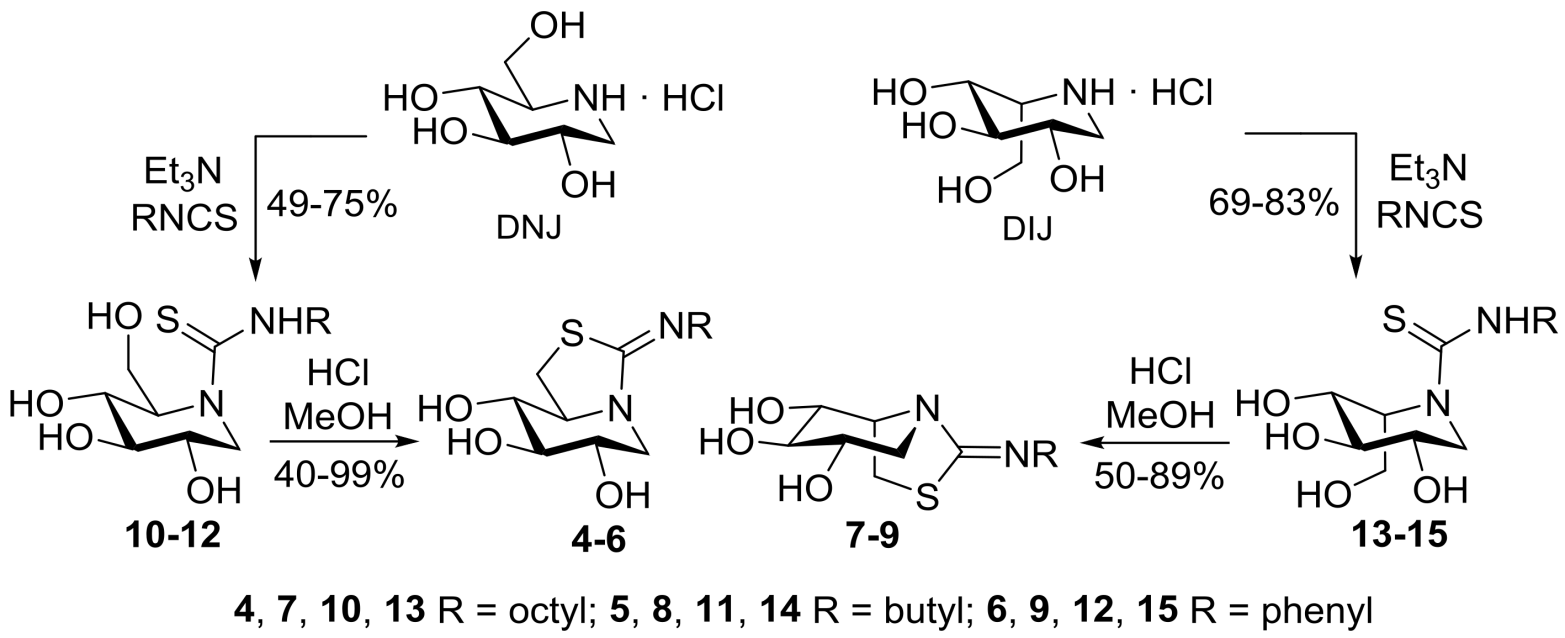
Synthesis of the new DNJ and DIJ mono- (**10**–**15**) and bicyclic sp^2^-iminosugars (**4**–**9**).

The new monocyclic thiourea derivatives of DNJ and DIJ **10**–**15** have been prepared by coupling reaction of the hydrochloride salts of the fully unprotected iminosugars with octyl, butyl, and phenyl isothiocyanates in the presence of triethylamine. Contrary to DNJ guanidines [78], the neutral thioureas are perfectly stable in water solution. After heating in methanol solution in the presence of hydrochloric acid, they undergo nucleophilic displacement of the primary hydroxyl by the thiocarbonyl sulfur to afford the target pyperidine-iminothiazolidine bicyclic derivatives **4**–**9** (Scheme 2). Note that compound **4** is, at the same time, the thio-analogue of **3** and the nonreducing homologue of **2**.

### 2.2. Inhibitory Properties against Commercial Enzymes

We first evaluated the inhibitory properties of the new nonreducing mono- and bicyclic sp^2^-iminosugars **4**–**15** against a panel of commercial glycosidases that included the α-glucosidases maltase (yeast maltase; α-Glcase1) and isomaltase (α-Glcase2) from *Saccharomyces cerevisiae* and amyloglucosidase (α-Glcase3) from *Aspergillus niger*, the β-glucosidases from almonds (β-Glcase1) and bovine liver (β-Glcase2), the α-galactosidase from green coffee beans (α-Galase), the β-galactosidase from *Escherichia coli* (β-Galase), and the α-mannosidase from Jack bean (α-Manase). The corresponding inhibition constant (*K*_i_) values are collected in Table 1. The data evidenced the superiority of compounds bearing *N′*-alkyl substituents as compared to phenyl conjugates in terms of both selectivity and inhibition potency towards the β-glucosidases. Within the *N′*-alkyl subgroup, the β-glucosidase inhibition selectivity and potency increased on going from monocyclic thioureas (*K*_i_ 227–1.3 μM for **10**, **11**, **13**, **14**) to bicyclic iminothiazolidines (*K*_i_ 185–0.045 μM for **4**, **5**, **7**, **8** against the almonds/bovine enzymes), the octyl derivatives affording the better results. When comparing the 5*N*,6*S*-(*N′*-octyliminomethylidene)-6-thio DNJ **4** (*K*_i_ 45 and 100 nM) and its DIJ epimer **7** (*K*_i_ 3.9 and 15 μM for the almonds and the mammalian enzyme, respectively), the former exhibits a 100-fold stronger inhibition potency towards the β-glucosidases. The better performing compound **4** was therefore selected for further evaluation of the inhibitory and chaperoning properties against human GCase in comparison with the previously reported candidates **1**, **2**, and **3**.

### 2.3. Inhibition Properties against Human GCase

Comparative assessment of the inhibition abilities of **1**–**4** against human lysosomal glycosidases in cell lysates (in vitro enzyme assay; see Experimental Section) confirmed the selectivity pattern previously observed for the commercial enzymes: whereas GCase was responsive to μM concentrations of the sp^2^-iminosugars, the activity of lysosomal α-glucosidase, α- and β-galactosidases, α- and β-mannosidases, and hexosaminidase was not affected at concentrations of up to 200 μM. The corresponding *K*_i_ values were determined at pH 7 and 5 using purified recombinant human GCase and 4-methylumbelliferone (4-MU)-conjugated α-d-glucopyranoside as the substrate (Table 2).

**Figure 3:**
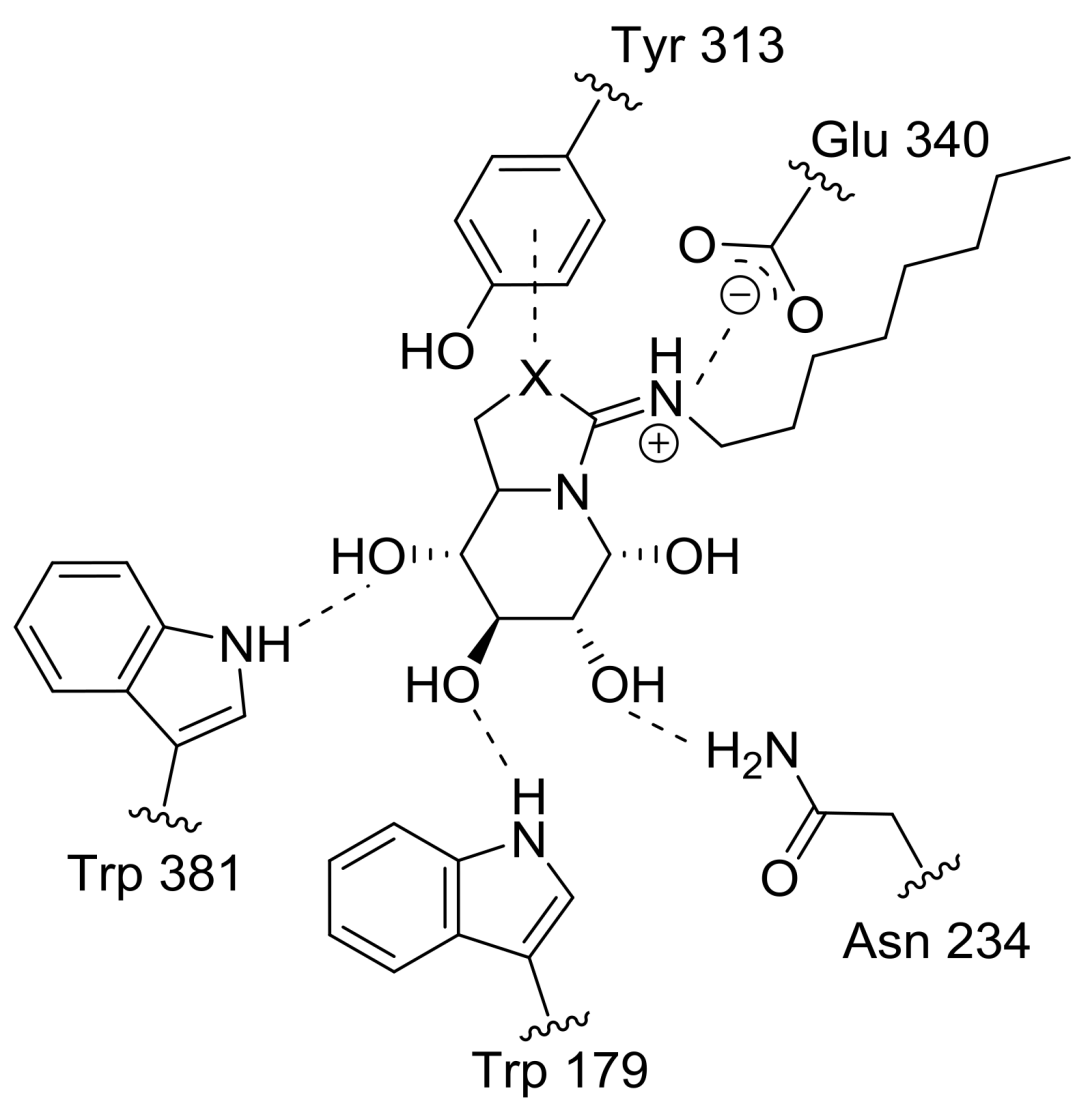
Salt bridge and hydrogen-bonding interactions of compounds **1** (X = O) and **2** (X = S) in the active site of human GCase as evidenced in the corresponding crystal structures (PDB codes 2XWD and 2XWE) [76]. The proposed sulfur–π interaction between the isothiourea S atom of **2** and the aromatic ring of tyrosine 313 is also depicted.

The data revealed a remarkable enhancement of the GCase inhibition potency upon replacement of O-6 in the five-membered ring of either the reducing (**1**) or nonreducing bicyclic sp^2^-iminosugar (**3**) into sulfur, reaching 58- or 52-fold in the case of **2** and 130- or 107-fold in the case of **4** at pH 7 or 5, respectively. The vital role played by sulfur in chemical and biological recognition and in drug development, either due to intramolecular effects or through promoting intermolecular interactions, is well recognized [81,82]. In our case, the p*K*_a_ values determined for the isourea derivatives **1** and **3** (8.9 and 9.3, respectively) indicated a slightly higher basicity as compared to the isothiourea counterparts **2** and **4** (8.3 in both cases). In any case, according to these data, the four sp^2^-iminosugar glycomimetics are expected to be protonated in the active site of GCase both at pH 7 and at pH 5. Indeed, the available X-ray structures of the GCase:**1** and GCase:**2** complexes show the presence of a similar salt bridge between the amidine segment—with the exocyclic double bond in the *E*-configuration—and a glutamic acid residue (Glu 340), discarding that interactions at this region of the molecule are responsible for the differences in affinity [76]. On the other hand, the oxygen/sulfur iso(thio)urea atom is found in close proximity (<3.5 Å) to a tyrosine residue (Tyr 313). Considering that noncovalent interactions of sulfur with π systems are significantly favored over oxygen [83,84], this may account for the experimental observations (Figure 3).

When comparing reducing and nonreducing pairs (i.e., **1** vs. **3** and **2** vs. **4**), the latter exhibited stronger GCase inhibition properties by about 10- and 20-fold, respectively. This is in agreement with the structural observation that enzyme:inhibitor complex formation for **1** and **2** implies the thermodynamically less stable β-anomer (Figure 3), with the corresponding enthalpy cost that is not compensated by productive interactions involving the anomeric hydroxyl. Regarding the effect of pH, all four sp^2^-iminosugars behaved as more potent GCase inhibitors at neutral than at acidic pH, a priori a favorable feature for pharmacological chaperone candidates: it means that the compound will bind more strongly to GCase at the ER, where stabilization of the correct folding is required, than at the lysosome, where dissociation of the GCase:chaperone complex is necessary to allow substrate processing. In this sense, it is worth mentioning that although GCase inhibition is generally considered a valuable indication for pharmacological chaperone drug candidates, a higher inhibitory potential does not necessarily translate into better activity enhancement abilities towards the target misfolding mutant enzyme. If displacement of the inhibitor from the enzyme:inhibitor complex is strongly disfavored, the enzyme would not be functional even though the compound may restore the proper folding and trafficking of mutant GCase variants [35].

### 2.4. GCase Chaperoning Capabilities

**Figure 4:**
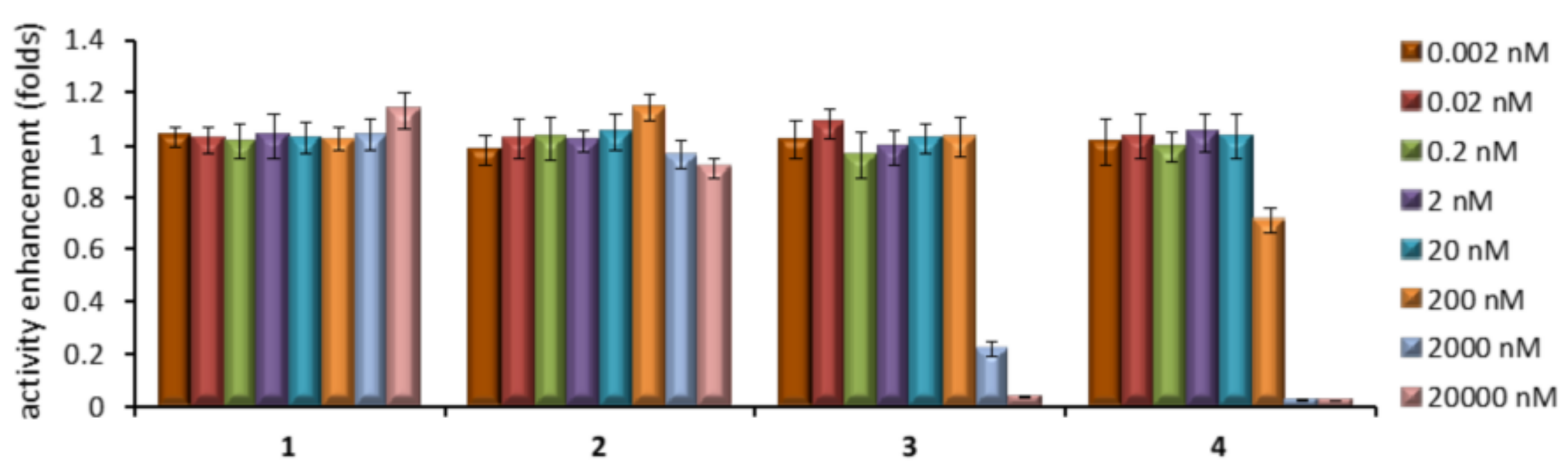
Effects of different concentrations of the sp^2^-iminosugar candidates **1**–**4** in the activity of GCase in cultured healthy human fibroblasts. Each bar represents the mean ± standard error (SEM) of three determinations each done in triplicate.

We first screened the effects of increasing concentrations of compounds **1**–**4** in GCase activity and cell viability using healthy human fibroblasts. For that purpose, cells were cultured for 5 days in the absence and in the presence of various concentrations of the compounds, then lysed, and the enzyme activity was determined using 4-methylumbelliferyl β-d-glucopyranoside as substrate. Higher GCase activities in the lysate indicate that larger amounts of the protein are present and that it is functional. None of the compounds had significant effect either on GCase activity (Figure 4) or on cell viability (data not shown) at concentrations up to 20 nM. At higher concentrations, the 1-deoxy derivatives **3** and **4** significantly inhibited GCase activity in the lysates, going down to virtually full inhibition at 20 μM. In contrast, GCase activity remained essentially unaltered in the presence of the reducing partners **1** and **2** over the whole range of concentrations (Figure 4).

**Figure 5:**
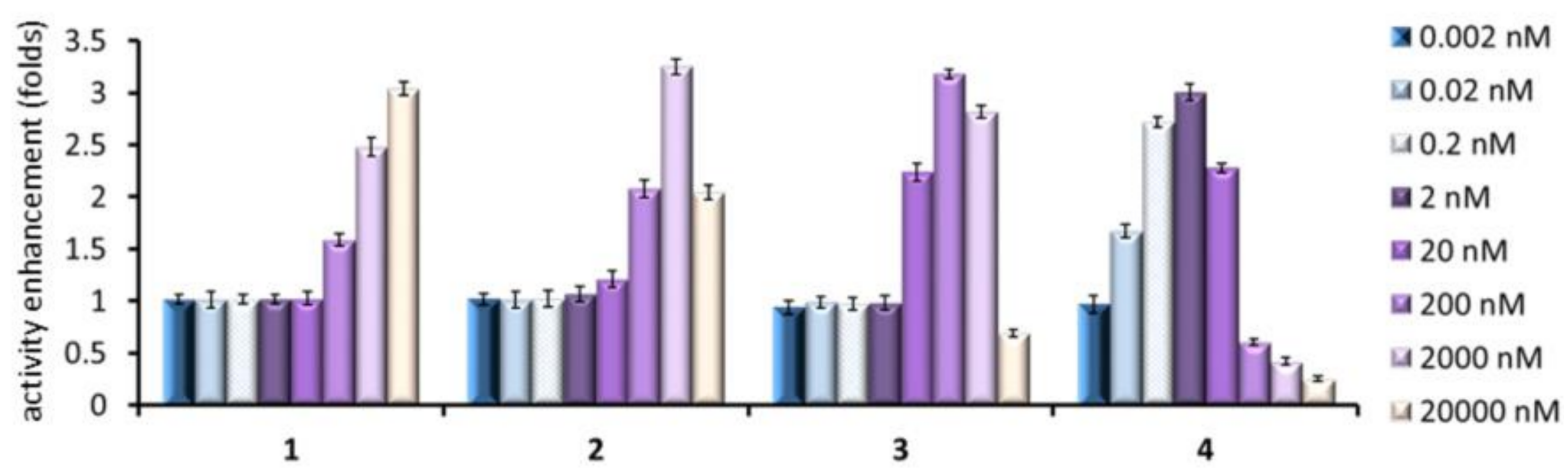
Mutant GCase activity enhancements in cultured human fibroblasts of N188S/G183W type 3 Gaucher patients promoted by the sp^2^-iminosugar candidates **1**–**4** at different concentrations. Each bar represents the mean ± standard error (SEM) of three determinations each done in triplicate.

To test the chaperoning capabilities, Gaucher fibroblasts from patients hosting the adult neuronopathic (type 3) Gaucher disease-associated N188S/G183W mutant GCase were employed. It is worth mentioning that the neuronopathic clinical subtypes of Gaucher disease remain currently at orphan status, given the inability of the recombinant enzyme used in enzyme replacement therapy or the glucosylceramide synthase inhibitor Zavesca^®^ used in substrate reduction therapy to cross the blood–brain barrier [6]. This mutation is located in the catalytic domain (domain III) of GCase and has been previously found to be responsive to sp^2^-iminosugar-type chaperones [19,51]. By following the aforementioned protocol for healthy fibroblasts, very significant mutant GCase activity enhancements (over 3-fold) were achieved with the whole set of compounds (Figure 5). Yet, the activity trend as a function of the chaperone concentration was starkly different between them. Thus, the nonreducing representatives **3** and **4** reached the maximum chaperoning effect at 200 nM and as low as 2 nM concentration, respectively, as compared with 20 and 2 μM for the reducing homologues **1** and **2**. Notably, compounds **3** and **4** exhibited unfavorable chaperone/inhibitor balance over 20 and 0.2 μM concentration, respectively—that is, they provoked a net decrease in the activity of the mutant enzyme compared to the control—which would result in aggravation of the disease.

**Figure 6:**
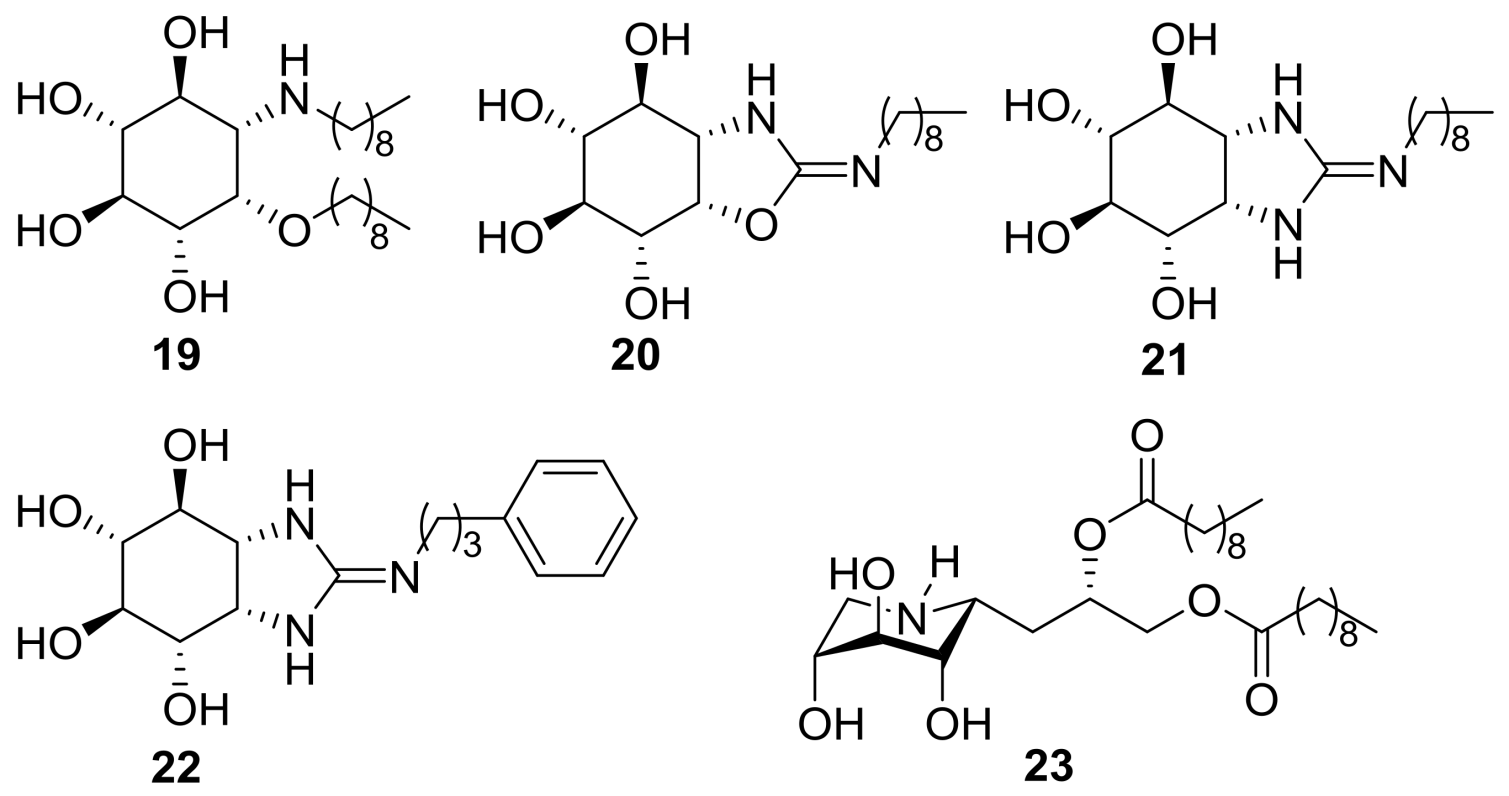
Structures of the pico- and nanomolar mutant GCase chaperones **19**–**23**.

Although full validation of the above results will require in situ GCase activity measurements [85,86], the collective data obtained from the in vitro assays strongly supports that the observed enzyme activity enhancements correspond indeed to the lysosomal and not to the cytoplasmatic GCase component, which is unaltered in Gaucher patients. To the best of our knowledge, the nonreducing isothiourea-type sp^2^-iminosugar **4**—which already promotes an over 70% N188S/G183W GCase activity enhancement at 20 pM concentration (300% at 2 nM) in patient fibroblasts—is among the most active chaperone candidates reported up to date for a neuronopathic Gaucher mutation. In this regard, **4** is only comparable to the aminocyclitol derivative (1*R*,2*S*,3*R*,4*S*,5*S*,6*R*)-5-(nonylamino)-6-(nonyloxy)cyclohexane-1,2,3,4-tetraol **19** (40% mutant GCase activity enhancement at 10 pM in homozygous L444P Gaucher lymphoblasts) [87], the related isourea and guanidine derivatives **20**–**22** (40–50% mutant GCase activity enhancement at 10 nM in homozygous L444P Gaucher lymphoblasts) [88], or the iminoxylytol glucosylceramide mimic **23** (100% mutant GCase activity enhancement at 50 nM in homozygous L444P Gaucher fibroblasts; Figure 6) [89].

Though the use of very low concentrations of chaperones is highly desirable due to the increase in selectivity for the target lysosomal enzyme and potentially lower side effects, their implementation in the clinic requires very strict dose control to avoid enzyme inhibition counterbalancing enzyme chaperoning, a problem already encountered with Galafold^®^ for the treatment of Fabry patients [90,91]. This can be particularly delicate in neuronopathic diseases, since relatively high concentrations in plasma and other organs are often necessary to achieve a therapeutic concentration of the drug in the central nervous system. The possibility to modulate the concentration range warranting a safe chaperone/inhibitor balance of the sp^2^-iminosugars by predefining their reducing or nonreducing character represents, therefore, an interesting feature in the optics of developing personalized chaperone therapies.

## 3. Materials and Methods

### 3.1. General Methods

Reagents and solvents were purchased from commercial sources and used without further purification. Optical rotations were measured with a JASCO P-2000 polarimeter (Jasco Analitica Spain, Madrid, Spain), using a sodium lamp (λ = 589 nm) at 22 °C in 1 cm or 1 dm tubes. Thin-layer chromatography (TLC) was carried out on aluminum sheets coated with Kieselgel 60 F245 (E. Merck, Darmstadt, Germany), with visualization by UV light and by charring with 10% H_2_SO_4_. Column chromatography was carried out on silica gel 60 (E. Merck, 230–400 mesh). IR spectra were recorded on a JASCO FTIR-410 device (Jasco Analitica Spain, Madrid, Spain). UV spectra were recorded on JASCO V-630 instrument (Jasco Analitica Spain, Madrid, Spain); unit for ε values: mM^−1^ cm^−1^. 1H (13C) NMR experiments were performed at 300 (75.5), 400 (100.6), and 500 (125.7) MHz. The corresponding spectra of the new compounds **4**–**15** are reproduced in the Supplementary Materials, Figures S1–S12. 1-D TOCSY as well as 2-D COSY and HMQC experiments were carried out to assist signal assignment. In the FABMS spectra, the primary beam consisted of Xe atoms with a maximum energy of 8 keV. The samples were dissolved in *m*-nitrobenzyl alcohol or thioglycerol as the matrices and the positive ions were separated and accelerated above a potential of 7 keV. NaI was added as cationizing agent. For ESI mass spectra, 0.1 pm sample concentrations were used, the mobile phase consisting of 50% aq MeCN at 0.1 mL min^−1^. Thin-layer chromatography was performed on precoated TLC plates, silica gel 30F-245, with visualization by UV light and also with 10% H_2_SO_4_ or 0.2% *w*/*v* cerium (IV) suphate-5% ammonium molybdate in 2 M H_2_SO_4_ or 0.1% ninhydrin in EtOH. Column chromatography was performed on Chromagel (silica 60 AC.C 70–200 μm). All compounds were purified to ≥95% purity as determined by elemental microanalysis results obtained on a CHNS-TruSpect^®^ Micro elemental analyzer (LECO, Madrid, Spain) at the Instituto de Investigaciones Químicas, (IIQ, Sevilla, Spain) from vacuum-dried samples. The analytical results for C, H, N, and S were within ±0.4 of the theoretical values.

### 3.2. Commercial Enzyme Inhibition Assays

Inhibition constant (*K*_i_) values were determined by spectrophotometrically measuring the residual hydrolytic activities of the glycosidases against the respective *o*- (for β-galactosidase from *E. coli*) or *p*-nitrophenyl α- or β-d-glycopyranoside (for other glycosidases) in the presence of the iminosugars. Each assay was performed in phosphate buffer or phosphate-citrate buffer (for α- or β-mannosidase and amyloglucosidase) at the optimal pH for the enzymes. The reactions were initiated by addition of the enzyme to a solution of the substrate in the absence or presence of various inhibitor concentrations. The mixture was incubated for 10–30 min at 37 °C or 55 °C (for amyloglucosidase) and the reaction was quenched by addition of 1 M Na_2_CO_3_. Reaction times were appropriate to obtain 10–20% conversion of the substrate in order to achieve linear rates. The absorbance of the resulting mixture was determined at 405 nm. Approximate value of *K*_i_ was determined from the slope of Dixon plots using a fixed concentration of substrate (around the *K*_m_ value for the different glycosidases) and various concentrations of inhibitor. Full *K*_i_ determinations and enzyme inhibition mode were determined from the slope of Lineweaver–Burk plots and double reciprocal analysis. Representative examples of Dixon and Lineweaver–Burk plots are shown in the Supplementary Materials, Figures S13 to S21.

### 3.3. Measurement of Purified Human GCase Inhibition Activities In Vitro

GCase activities were determined as above using purified human GCase, obtained from Genzyme (Genzyme Japan, Tokyo, Japan) in 0.1 M citrate buffer at pH 5 or pH 7 and 4-methylumbelliferone (4-MU)-conjugated β-d-glucopyranoside as the substrate. The reactions were terminated by adding 0.2 mL of 0.2 M glycine sodium hydroxide buffer (pH 10.7). The liberated 4-methylumbelliferone was measured in a black-well plate with a Perkin Elmer Luminescence Spectrometer, Waltham, MA, USA (excitation wavelength: 340 nm; emission: 460 nm).

### 3.4. Cell Cultures, Chaperone Tests, and Toxicity Assays

Normal and Gaucher disease patients’ skin fibroblasts were cultured in Dulbecco modified Eagle’s medium (WAKO, Richmond, VA, USA) supplemented with 10% fetal bovine serum (Nichirei Biosci. Inc., Tokyo, Japan) at 37 °C in 5% CO_2_. For measurement of chaperone activities, fibroblasts were plated onto 35 mm dishes at 50,000 cells per dish. After 24 h incubation, the medium with the indicated concentrations of compound was applied and cells were cultured for 96 h. Then, the lysates in 0.1% Triton-X100/distilled H_2_O were collected from the cells and assayed for lysosomal GCase as described above. To measure the cytotoxic effect of the compounds, normal human fibroblasts were plated onto 35 mm dishes at 15,000 cells per dish and incubated overnight. Then, the medium was changed with or without compounds. After 24 h incubation, the supernatant of the cells was collected and measured by the lactate dehydrogenase assay (WAKO, Richmond, VA, USA). All the cell lines used were tested for mycoplasma contamination before conducting the chaperone and toxicity assays.

### 3.5. Statistical Analysis

All results are expressed as mean ± SD of three independent experiments, each conducted in triplicate. The measurements were statistically analyzed using the Student’s *t* test for comparing two groups. The level of significance was set at *p* < 0.05.

### 3.6. General Procedure for the Synthesis of the DNJ-Related Thioureas ***10**–**12***

To a solution of 1-deoxynojirimicin hydrochloride (102 mg, 0.63 mmol) and Et_3_N (0.1 mL, 0.75 mmol, 1.2 eq) in pyridine (9.1 mL), octyl, butyl of phenyl isothiocyanate (1.1 eq) was added. The mixture was stirred at rt for 18 h and the solvent was removed. The resulting residue was coevaporated several times with toluene and purified by column chromatography using the eluting solvent indicated in each case.

*1-Deoxy-N-(N′-octylthiocarbamoyl)nojirimicin* (**10**). Column chromatography: 100:10:1 → 70:10:1 DCM-MeOH-H_2_O; yield: 156 mg (75%); [α]_D_ −171.6 (*c* 1.0, CH_3_OH); *R*_f_ 0.60 (40:10:1 DCM-MeOH-H_2_O); UV (CH_3_OH) 248 nm (ε_mM_ 12.8); ^1^H NMR (500 MHz, CD_3_OD) δ 4.55 (d, 1 H, *J*_1a,1b_ = 14.6 Hz, H-1a), 4.37 (m, 1 H, H-5), 3.89 (dd, 1 H, *J*_6a,6b_ = 11.3 Hz, *J*_5,6a_ = 4.0 Hz, H-6a), 3.85 (dd, 1 H, *J*_5,6b_ = 7.7 Hz, H-6b), 3.73 (m, 1 H, H-2), 3.57 (m, 4 H, H-4, H-3, CH_2_N), 3.50 (dd, 1 H, *J*_1b,2_ = 3.5 Hz, H-1b), 1.61 (m, 2 H, CH_2_), 1.32 (m, 10 H, CH_2_), 0.90 (t, 3 H, ^3^*J*_H,H_ = 7.1 Hz, CH_3_).; ^13^C NMR (125.7 MHz, CD_3_OD) δ 186.5 (CS), 75.1 (C-3), 73.7 (C-2), 71.1 (C-4), 66.6 (C-5), 62.4 (C-6), 48.3 (C-1), 47.1 (CH_2_N), 32.9, 30.4, 30.3, 30.1, 28.0 (CH_2_), 23.6 (*C*H_2_CH_3_), 14.3 (CH_3_); FABMS: *m*/*z* 357 (100, [M + Na]^+^), 335 (10, [M + H]^+^); Anal. Calcd. for C_15_H_30_N_2_O_4_S: C, 53.86; H, 9.04; N, 8.38; S, 9.59; found: C, 53.72; H, 8.97; N, 8.32; S, 9.40.

*1-Deoxy-N-(N′-butylthiocarbamoyl)nojirimicin* (**11**). Column chromatography: 90:10:1 DCM-MeOH-H_2_O; yield: 109 mg (62%); [α]_D_ = −206.0 (*c* 1.0, MeOH); *R*_f_ = 0.29 (DCM-MeOH-H_2_O 70:10:1). UV (MeOH): 247 nm (ε_mM_ 13.0); ^1^H NMR (500 MHz, CD_3_OD) δ 4.54 (d, 1 H, *J*_1a,1b_ = 14.7 Hz, H-1a), 4.35 (m, 1 H, H-5), 3.89 (dd, 1 H, *J*_6a,6b_ = 11.3 Hz, *J*_5,6a_ = 3.9 Hz, H-6a), 3.84 (dd, 1 H, *J*_5,6b_ = 7.8 Hz, H-6b), 3.73 (m, 1 H, H-2), 3.57 (m, 3 H, H-4, CH_2_N), 3.52 (t, 1 H, *J*_2,3_ = *J*_3,4_ = 6.4 Hz, H-3), 3.50 (dd, 1 H, *J*_1b,2_ = 3.6 Hz, H-1b), 1.59 (m, 2 H, CH_2_), 1.39 (m, 2 H, CH_2_), 0.95 (t, 3 H, ^3^*J*_H,H_ = 7.4 Hz, CH_3_); ^13^C NMR (125.7 MHz, CD_3_OD) δ 186.4 (CS), 75.2 (C-3), 73.8 (C-2), 70.9 (C-4), 66.6 (C-5), 62.3 (C-6), 48.3 (C-1), 46.7 (CH_2_N), 32.3 (CH_2_), 21.1 (*C*H_2_CH_3_), 14.2 (CH_3_); FABMS: *m*/*z* 301 (100, [M + Na]^+^), 279 (10, [M + H]^+^); Anal. Calcd. for C_11_H_22_N_2_O_4_S: C, 47.46; H, 7.97; N, 10.06; S, 11.52; found: C, 47.43; H, 7.77; N, 9.84; S, 11.17.

*1-Deoxy-N-(N′-phenylthiocarbamoyl)nojirimicin* (**12**). Column chromatography: 100:10:1 → 90:10:1 DCM-MeOH-H_2_O; yield: 92 mg (49%); [α]_D_ = −196.7 (*c* 1.0, MeOH); *R*_f_ = 0.29 (70:10:1 DCM-MeOH-H_2_O); UV (MeOH): 255 nm (ε_mM_ 11.3); ^1^H NMR (300 MHz, CD_3_OD) δ 7.35–7.09 (m, 5 H, Ph), 4.72 (d, 1 H, *J*_1a,1b_ = 14.6 Hz, H-1a), 4.61 (m, 1 H, H-5), 3.98 (d, 2 H, *J*_5,6_ = 5.8 Hz, H-6), 3.81 (m, 1 H, H-2), 3.69 (m, 1 H, H-4), 3.63 (m, 2 H, H-3, CH_2_N), 3.62 (dd, 1 H, *J*_1b,2_ = 3.8 Hz, H-1b); ^13^C NMR (75.5 MHz, CD_3_OD) δ 186.4 (CS), 141.9–125.5 (Ph), 74.8 (C-3), 73.6 (C-2), 71.0 (C-4), 66.9 (C-5), 62.4 (C-6), 50.9 (C-1); FABMS: *m*/*z* 321 (90, [M + Na]^+^), 299 (20, [M + H]^+^); HRFABMS: *m*/*z* 321.0893; Calcd. for C_11_H_18_N_2_O_4_NaS: 321.0885.

### 3.7. General Procedure for the Synthesis of the DNJ-Related Bicyclic Isothioureas ***4**–**6***

To a solution of the corresponding thioureido derivative **10**–**12** (0.27 mmol) in MeOH (8.5 mL), concentrated HCl was added until pH 1 (1–2 drops). The reaction mixture was stirred at room temperature for 12 h and the solvent was removed. The resulting residue was coevaporated several times with MeOH until neutral pH and purified by column chromatography using the eluent indicated in each case.

*1-Deoxy-6-thio-5-N,6-S-(N′-octyliminomethylidene)nojirimycin* (**4**). Column chromatography: 70:10:1 → 40:10:1 DCM-MeOH-H_2_O; yield: 85 mg (quantitative); [α]_D_ + 11.7 (*c* 0.96, MeOH); *R*_f_ 0.34 (40:10:1 DCM-MeOH-H_2_O); ^1^H NMR (500 MHz, CD_3_OD) δ 4.13 (dd, 1 H, *J*_1a,1b_ = 13.1 Hz, *J*_1a,2_ = 5.6 Hz, H-1a), 3.96 (m, 1 H, H-5), 3.72 (dd, 1 H, *J*_6a,6b_ = 11.4 Hz, *J*_5,6a_ = 7.7 Hz, H-6a), 3.52 (m, 1 H, H-2), 3.46 (dd, 1 H, *J*_5,6a_ = 7.5 Hz, H-6b), 3.37 (m, 4 H, H-3, H-4, CH_2_N), 3.00 (dd, 1 H, *J*_1b,2_ = 11.1 Hz, H-1b), 1.68 (m, 2 H, CH_2_), 1.33 (m, 10 H, CH_2_), 0.91 (t, 3 H, ^3^*J*_H,H_ = 7.1 Hz, CH_3_); ^13^C NMR (125.7 MHz, CD_3_OD) δ 171.3 (CN), 78.8 (C-3), 73.8 (C-4), 69.5 (C-2), 69.0 (C-5), 50.8 (CH_2_N), 48.7 (C-1), 32.9 (CH_2_), 31.9 (C-6), 30.3, 30.2, 27.7, 23.7 (CH_2_), 14.4 (CH_3_); FABMS: *m*/*z* 317 (100, [M + H]^+^); HRFABMS: *m*/*z* 317.1910; calcd. for C_15_H_29_N_2_O_3_S 317.1899.

*1-Deoxy-6-thio-5-N,6-S-(N′-butyliminomethylidene)nojirimycin* (**5**). Column chromatography: 60:10:1 → 50:10:1 DCM-MeOH-H_2_O; yield: 71 mg (quantitative); [α]_D_ = +13.7 (*c* 1.2, MeOH); *R*_f_ = 0.31 (40:10:1 DCM-MeOH-H_2_O); ^1^H NMR (500 MHz, CD_3_OD) δ 4.14 (dd, 1 H, *J*_1a,1b_ = 13.1 Hz, *J*_1a,2_ = 5.6 Hz, H-1a), 3.97 (m, 1 H, H-5), 3.72 (dd, 1 H, *J*_6a,6b_ = 11.4 Hz, *J*_5,6a_ = 7.7 Hz, H-6a), 3.52 (m, 1 H, H-2), 3.47 (dd, 1 H, *J*_5,6a_ = 7.4 Hz, H-6b), 3.37 (m, 4 H, H-3, H-4, CH_2_N), 3.00 (dd, 1 H, *J*_1b,2_ = 11.0 Hz, H-1b), 1.66 (m, 2 H, CH_2_), 1.40 (m, 2 H, CH_2_), 0.97 (t, 3 H, ^3^*J*_H,H_ = 7.4 Hz, CH_3_); ^13^C NMR (125.7 MHz, CD_3_OD): δ 171.5 (CN), 78.8 (C-3), 73.8 (C-4), 69.5 (C-2), 69.1 (C-5), 50.5 (CH_2_N), 48.8 (C-1), 32.3 (CH_2_), 31.9 (C-6), 20.8 (CH_2_), 13.9 (CH_3_). FABMS: *m*/*z* 261 (100, [M + H]^+^). HRFABMS: *m*/*z* 383.1082; Calcd. for C_11_H_20_N_2_O_3_NaS 283.1092; Anal. Calcd. for C_11_H_20_N_2_O_3_S: C, 50.75; H, 7.74; N, 10.76; S, 12.32; found: C, 50.41; H, 7.55; N, 10.37; S, 12.01.

*1-Deoxy-6-thio-5-N,6-S-(N′-phenyliminomethylidene)nojirimycin* (**6**). Column chromatography: 100:10:1 → 20:10:1 DCM-MeOH-NH_4_OH; yield: 30 mg (40%); [α]_D_ = +3.0 (*c* 0.5, MeOH); *R*_f_ = 0.50 (50:10:1 DCM-MeOH-H_2_O); ^1^H NMR (300 MHz, CD_3_OD) δ 7.28–6.88 (m, 5 H, Ph), 4.29 (dd, 1 H, *J*_1a,1b_ = 12.8 Hz, *J*_1a,2_ = 5.5 Hz, H-1a), 3.53 (m, 2 H, H-5, H-2), 3.39 (dd, 1 H, *J*_6a,6b_ = 10.9 Hz, *J*_5,6a_ = 6.7 Hz, H-6a), 3.33 (m, 2 H, H-3, H-4), 3.14 (dd, 1 H, *J*_5,6a_ = 6.3 Hz, H-6b), 2.74 (dd, 1 H, *J*_1b,2_ = 10.8 Hz, H-1b); ^13^C NMR (75.5 MHz, CD_3_OD) δ 162.7 (CN), 152.7–123.3 (Ph), 79.8 (C-3), 74.2 (C-4), 70.0 (C-2), 66.0 (C-5), 48.9 (C-1); FABMS: *m*/*z* 281 (30, [M + H]^+^). HRFABMS: *m*/*z* 281.0955; calcd. for C_13_H_17_N_2_O_3_S 281.0960; Anal. Calcd. for C_13_H_16_N_2_O_3_S: C, 55.70; H, 5.75; N, 9.99; S, 11.44; found: C, 55.39; H, 5.53; N, 9.78; S, 11.17.

### 3.8. General Procedure for the Synthesis of the DIJ-Related Thioureas ***13**–**15***

To a solution of DIJ hydrochloride (100 mg, 0.613 mmol) in pyridine (5 mL), Et_3_N (100 μL, 0.735 mmol) and octyl, butyl of phenyl isothiocyanate (0.674 mmol) were added. The mixture was stirred at rt for 18 h and concentrated. The resulting residue coevaporated several times with toluene and purified by column chromatography using the eluting solvent indicated in each case.

*N-(N′-Octylthiocarbamoyl)-1-deoxy-l-idonojirimycin* (**13**). Column chromatography (3:1 EtOAc-petroleum ether → EtOAc → 20:1 EtOAc-EtOH → 45:5:3 EtOAc-EtOH-H_2_O); yield: 170 mg (83%); *R*_f_ 0.48 (45:5:3 EtOAc-EtOH-H_2_O); [α]_D_ −102.1 (*c* 0.87, EtOH); UV (EtOH) λ_max_ 251 nm (ε_mM_ 8.6); ^1^H NMR (300 MHz, 9:1 acetone-*d*_6_-D_2_O, 313 K) δ 4.94 (dd, 1 H, *J*_1a,1b_ = 13.6 Hz, *J*_1a,2_ = 4.5 Hz, H-1a), 4.85 (m, 1 H, H-5), 4.00 (dd, 1 H, *J*_6a,6b_ = 11.5 Hz, *J*_5,6a_ = 3.9 Hz, H-6a), 3.87 (dd, 1 H, *J*_5,6b_ = 9.3 Hz, H-6b), 3.54 (m, 2 H, C*H*_2_NH_2_), 3.52 (m, 3 H, H-2, H-3, H-4), 2.93 (dd, 1 H, *J*_1b,2_ = 10.6 Hz, H-1b), 1.57 (m, 2 H, C*H*_2_CH_2_NH_2_), 1.27 (m, 10 H, CH_2_), 0.86 (t, 3 H, CH_3_);^13^C NMR (75.5 MHz, 9:1 acetone-*d*_6_-D_2_O, 313 K) δ 185.6 (CS), 76.3 (C-3), 71.7 (C-4), 70.4 (C-2), 61.6 (C-5), 58.1 (C-6), 48.3 (C-1), 46.6 (CH_2_NH_2_), 32.4, 27.6, 23.2 (CH_2_), 14.2 (CH_3_); ESIMS *m*/*z* 357 ([M + Na]^+^). Anal. Calcd. for C_15_H_30_N_2_O_4_S: C, 53.86; H, 9.04; N, 8.38; S, 9.59; found: 53.64; H, 8.873; N, 8.10; S, 9.21.

*N-(N′-Butylthiocarbamoyl)-1-deoxy-l-idonojirimycin* (**14**). Column chromatography (2:1 EtOAc-cyclohexane → EtOAc → 20:1 EtOAc-EtOH → 45:5:3 EtOAc-EtOH-H_2_O); yield: 141 mg (83%); *R*_f_ 0.39 (45:5:3 EtOAc-EtOH-H_2_O); [α]_D_ + 118.8 (*c* 0.69, EtOH); UV (EtOH) λ_max_ 250 nm (ε_mM_ 10.8).^1^H NMR (500 MHz, 9:1 acetone-*d*_6_/D_2_O, 313 K) δ 4.96 (dd, 1 H, *J*_1a,1b_ = 13.3 Hz, *J*_1a,2_ = 4.3 Hz, H-1a), 4.82 (m, 1 H, H-5), 4.00 (dd, 1 H, *J*_6a,6b_ = 11.5 Hz, *J*_5,6a_ = 3.8 Hz, H-6a), 3.87 (dd, 1 H, *J*_5,6b_ = 9.3 Hz, H-6b), 3.57 (m, 2 H, C*H*_2_NH_2_), 3.52 (dd, 1 H, *J*_3,4_ = 9.4 Hz, *J*_4,5_ = 5.9 Hz, H-4), 3.46 (m, 1 H, H-3), 3.40 (m, 1 H, H-2), 2.92 (dd, 1 H, *J*_1b,2_ = 10.8 Hz, H-1b), 1.56 (m, 2 H, C*H*_2_CH_2_NH_2_), 1.33 (m, 2 H, C*H*_2_CH_3_), 0.89 (t, 3 H, CH_3_); ^13^C NMR (500 MHz, 9:1 acetone-*d*_6_/D_2_O, 313 K) δ 185.7 (CS), 76.4 (C-3), 71.8 (C-4), 70.4 (C-2), 61.6 (C-5), 58.1 (C-6), 48.3 (C-1), 46.3 (CH_2_NH_2_), 31.8, 20.7 (CH_2_), 14.1 (CH_3_); ESIMS *m*/*z* 301 ([M + Na]^+^); Anal. Calcd. for C_11_H_22_N_2_O_4_S: C, 47.46; H, 7.97; N, 10.06; S, 11.52; found: C, 47.21; H, 7.901; N, 9.74; S, 11.18.

*N-(N′-Phenylthiocarbamoyl)-1-deoxy-l-idonojirimycin* (**15**). Column chromatography (3:1 EtOAc-petroleum ether → EtOAc → 20:1 EtOAc-EtOH → 45:5:3 EtOAc-EtOH-H_2_O); Yield: 146 mg (80%); *R*_f_ 0.47 (45:5:3 EtOAc-EtOH-H_2_O); [α${]}_{D}^{20}$ + 56.1 (*c* 1.0, MeOH); UV (MeOH) λ_max_ 256 nm (ε_mM_ 11.3); ^1^H NMR (500 MHz, CD_3_OD, 313 K) δ 7.32–7.10 (m, 5 H, Ph), 5.03 (m, 1 H, H-5), 4.97 (d, 1 H, *J*_1a,1b_ = 13.5 Hz, H-1a), 4.09 (dd, 1 H, *J*_6a,6b_ = 11.5 Hz, *J*_5,6a_ = 3.6 Hz, H-6a), 3.96 (dd, 1 H, *J*_5,6b_ = 9.8 Hz, H-6b), 3.64 (dd, 1 H, *J*_3,4_ = 9.6 Hz, *J*_4,5_ = 6.3 Hz, H-4), 3.49 (m, 2 H, H-2, H-3), 3.04 (dd, 1 H, *J*_1b,2_ = 10.6 Hz, H-1b);^13^C NMR (125.7 MHz, CD_3_OD, 313 K) δ 191.0 (CS), 129.5–125.6(Ph), 76.5 (C-3), 71.9 (C-4), 70.9 (C-2), 63.5 (C-5), 58.5 (C-6), 48.3 (C-1; ESIMS *m*/*z* 297 ([M − H]^−^); Anal. Calcd. for C_13_H_18_N_2_O_4_S: C, 52.33; H, 6.08; N, 9.39; S, 10.75; found: C, 52.05; H, 5.922; N, 9.17; S, 10.39.

### 3.9. General Procedure for the Synthesis of the DIJ-Related Bicyclic Isothioureas ***7**–**9***

To a solution of the corresponding thioureido derivative **13**–**15** (0.30 mmol) in MeOH (12 mL), concentrated HCl was added until pH 1 (1–2 drops). The reaction mixture was stirred at 65–85 °C for 2–8 h and the solvent was removed (precise conditions are indicated hereinafter). The resulting residue was coevaporated several times with MeOH until neutral pH and purified by column chromatography using the eluent indicated in each case.

*5-N,6-S-(N′-Octyliminomethylidene)-6-thio-1-deoxy-l-idonojirimycin* (**7**). Temperature: 85 °C; time of reaction: 8 h; column chromatography (3:1 EtOAc-petroleum ether → EtOAc → 20:1 EtOAc-EtOH → 45:5:3 EtOAc-EtOH-H_2_O); Yield: 85 mg (89%); *R*_f_ 0.12 (45:5:3 EtOAc-EtOH-H_2_O). [α]_D_ + 19.9 (*c* 1.0, MeOH); ^1^H NMR (400 MHz, CD_3_OD) δ 4.60 (td, 1 H, *J*_5,6a_ = *J*_5,6b_ = 8.2 Hz, *J*_4,5_ = 1.9 Hz, H-5), 4.07 (dd, 1 H, *J*_1a,1b_ = 13.8 Hz, *J*_1a,2_ = 1.8 Hz, H-1a), 4.01 (m, 1 H, H-3), 3.90 (m, 1 H, H-2), 3.79 (m, 1 H, H-4), 3.64 (dd, 1 H, *J*_1b,2_ = 2.0 Hz, H-1b), 3.62 (m, 2 H, H-6a, H-6b), 3.36 (td, 2 H, C*H*_2_NH); ^13^C NMR (100.6 MHz, CD_3_OD) δ 172.9 (CN), 72.6 (C-4), 69.8 (C-2), 68.8 (C-3), 65.7 (C-5), 49.8 (CH_2_NH), 48.4 (C-1), 30.2 (CH_2_), 29.2 (C-6), 27.5, 23.7 (CH_2_), 14.4 (CH_3_); ESIMS *m*/*z* 317 ([M + H]^+^); Anal. Calcd. for C_15_H_28_N_2_O_3_S: C, 56.93; H, 8.92; N, 8.85; S, 10.30; found: C, 56.98; H, 8.916; N, 8.68; S, 10.32.

*5-N,6-S-(N′-Butyliminomethylidene)-6-thio-1-deoxy-l-idonojirimycin* (**8**). Temperature: 85 °C; time of reaction: 8 h; column chromatography (3:1 EtOAc-petroleum ether → EtOAc → 20:1 EtOAc-EtOH → 45:5:3 EtOAc-EtOH-H_2_O); yield: 58 mg (74%); *R*_f_ 0.15 (45:5:3 EtOAc-EtOH-H_2_O); [α]_D_ + 24.0 (*c* 1.0, EtOH); ^1^H NMR (500 MHz, 9:1 acetone-*d*_6_-D_2_O) δ 4.62 (ddd, 1 H, *J*_5,6a_ = 9.2 Hz, *J*_5,6b_ = 7.6 Hz, *J*_4,5_ = 1.9 Hz, H-5), 4.51 (dd, 1 H, *J*_1a,1b_ = 14.0 Hz, *J*_1a,2_ = 1.8 Hz, H-1a), 4.05 (m, 1 H, H-3), 4.03 (m, 1 H, H-2), 3.79 (m, 1 H, H-4), 3.74 (dd, 1 H, *J*_6a,6b_ = 10.9 Hz, H-6a), 3.68 (dd, 1 H, H-6b), 3.60 (dd, 1 H, *J*_1b,2_ = 1.7 Hz, H-1b), 3.36 (td, 2 H, C*H*_2_NH), 1.67 (m, 2 H, C*H*_2_CH_2_NH), 1.38 (m, 2 H, C*H*_2_CH_3_), 0.90 (t, 3 H, CH_3_); ^13^C NMR (125.7 MHz, 9:1 acetone-*d*_6_-D_2_O) δ 171.8 (CN), 72.3 (C-4), 69.3 (C-2), 68.0 (C-3), 65.5 (C-5), 49.1 (CH_2_NH), 48.5 (C-1), 31.8 (CH_2_), 29.0 (C-6), 20.3 (CH_2_), 13.9 (CH_3_); ESIMS *m*/*z* 283 ([M + Na]^+^); Anal. Calcd. for C_11_H_20_N_2_O_3_S: C, 50.75; H, 7.74; N, 10.76; S, 12.32; found: C, 50.42; H, 7.455; N, 10.39; S, 12.92.

*5-N,6-S-(N′-Phenyliminomethylidene)-6-thio-1-deoxy-l-idonojirimycin* (**9**). Temperature: 65 °C; time of reaction: 2 h; column chromatography (3:1 EtOAc-petroleum ether → EtOAc → 20:1 EtOAc-EtOH → 45:5:3 EtOAc-EtOH-H_2_O); yield: 42 mg (50%); *R*_f_ 0.55 (45:5:3 EtOAc-EtOH-H_2_O). [α]_D_ + 50.9 (*c* 1.0, MeOH); ^1^H NMR (300 MHz, CD_3_OD) δ 7.28–6.92 (m, 5 H, Ph), 4.15 (dd, 1 H, *J*_1a,1b_ = 13.5 Hz, *J*_1a,2_ = 1.4 Hz, H-1a), 4.07 (m, 1 H, H-3), 3.9 (ddd, 1 H, *J*_5,6a_ = 10.5 Hz, *J*_5,6b_ = 7.2 Hz, *J*_4,5_ = 1.6 Hz, H-5), 3.89 (m, 1 H, H-2), 3.74 (m, 1 H, H-4), 3.41 (t, 1 H, *J*_5,6a_ = *J*_6a,6b_ = 10.5 Hz, H-6a), 3.27 (dd, 1 H, *J*_1a,1b_ = 13.9 Hz, *J*_1b,2_ = 1.9 Hz, H-1b), 3.10 (dd, 1 H, H-6b); ^13^C NMR (75.5 MHz, CD_3_OD) δ 165.8 (CN), 152.2–123.7 (Ph), 71.5 (C-4), 69.8 (C-2), 69.4 (C-3), 61.8 (C-5), 48.0 (C-1), 28.4 (C-6); ESIMS *m*/*z* 281 ([M + H]^+^); Anal. Calcd. for C_13_H_16_N_2_O_3_S: C, 55.70; H, 5.75; N, 9.99; S, 11.14; found: C, 55.56; H, 5.604; N, 9.88; S, 11.32.

## Figures and Tables

**Table 1:** Glycosidase inhibitory activities (*K*_i_, μM) of the new DNJ and DIJ isothiourea (**4**–**9**) and thiourea (**10**–**15**) derivatives. Values represent the mean ± SD (three independent determinations). Inhibition was competitive in all the cases.

Comp.	α-Glcase1	α-Glcase2	α-Glcase3	β-Glcase1	β-Glcase2	α-Galase	β-Galase	α-Manase
**4**	n.i. ^1^	n.i.	n.i.	0.045 ^2^	0.1 ± 0.02	n.i.	n.i.	n.i.
**5**	n.i.	n.i.	n.i.	1.1 ± 0.1	5.8 ± 0.5	n.i.	n.i.	n.i.
**6**	406 ± 20	n.i.	44 ± 3	48 ± 4	15 ± 1	n.i.	n.i.	n.i.
**7**	n.i.	n.i.	n.i.	3.9 ± 0.3	15 ± 2	504 ± 32	n.i.	n.i.
**8**	n.i.	n.i.	n.i.	19 ± 9	185 ± 14	n.i.	n.i.	n.i.
**9**	262 ± 15	n.i.	n.i.	309 ± 19	255 ± 20	772 ± 35	n.i.	n.i.
**10**	481 ± 27	116 ± 8	129 ± 10	1.3 ± 0.1	1.3 ± 0.1	n.i.	n.i.	n.i.
**11**	568 ± 30	121 ± 9	213 ± 18	20 ± 2	12.7 ± 1	847 ± 42	n.i.	n.i.
**12**	60 ± 4	10 ± 1	18 ± 2	23 ± 3	71 ± 8	122 ± 9	n.i.	294 ± 18
**13**	n.i.	n.i.	n.i.	227 ± 13	2.3 ± 0.2	60 ± 15	n.i.	n.i.
**14**	n.i.	n.i.	n.i.	51 ± 13	11 ± 1	n.i.	n.i.	n.i.
**15**	293 ± 18	443 ± 22	271 ± 17	294 ± 18	66 ± 15	77 ± 16	n.i.	n.i.

^1^ No inhibition detected at 1 mM. ^2^ Error: ±0.002.

**Table 2:** Inhibition constant (*K*_i_) values (μM) for the selected sp^2^-iminosugar chaperone candidates **1**–**4** against human β-glucocerebrosidase (GCase).^1^ Inhibition was competitive in all the cases.

pH	1	2	3	4
7	15.1 ± 0.97	0.26 ± 0.013	1.7 ± 0.066	0.013 ± 0.0011
5	54.4 ± 0.38	1.05 ± 0.068	6.3 ± 0.16	0.059 ±0.0035

^1^ Purified recombinant enzyme obtained from Sanofi Genzyme, Cambridge, MA, USA (Cerezyme^®^).

## Supplementary Materials

The following are available online. Figures S1–S12: ^1^H and ^13^C NMR spectra of the new compounds **4**–**15**, and Figures S13–S21: selected Dixon and Lineweaver-Burk plots for *K*i determinations.
