# Supplementary material for: Probing the Inhibitor versus Chaperone Properties of sp2-Iminosugars towards Human β-Glucocerebrosidase: A Picomolar Chaperone for Gaucher Disease
Source: Molecules. 2018 Apr 17;23(4):927. doi: 10.3390/molecules23040927 (PMC6017062; doi:10.3390/molecules23040927)
Supplement: Supplementary file 1 [file molecules-23-00927-s001.pdf]

Probing the inhibitor versus chaperone properties of sp^2-iminosugars towards human β-glucocerebrosidase: A picomolar chaperone for Gaucher disease

Teresa Mena-Barragán[1], M. Isabel García-Moreno[1], Alen Sevšek[2], Tetsuya Okazaki,[3] Eiji Nanba,[4] Katsumi Higaki[4,*], Nathaniel I. Martin,[2,*] Roland J. Pieters[2,*], José Manuel García Fernández[5,*], Carmen Ortiz Mellet[1,*]

Supplementary Information

List of contents

S1 to S12 NMR spectra of compounds **4**-**15**.

S13 to S33 Dixon and Lineweaver-Burk plots for K_i determination

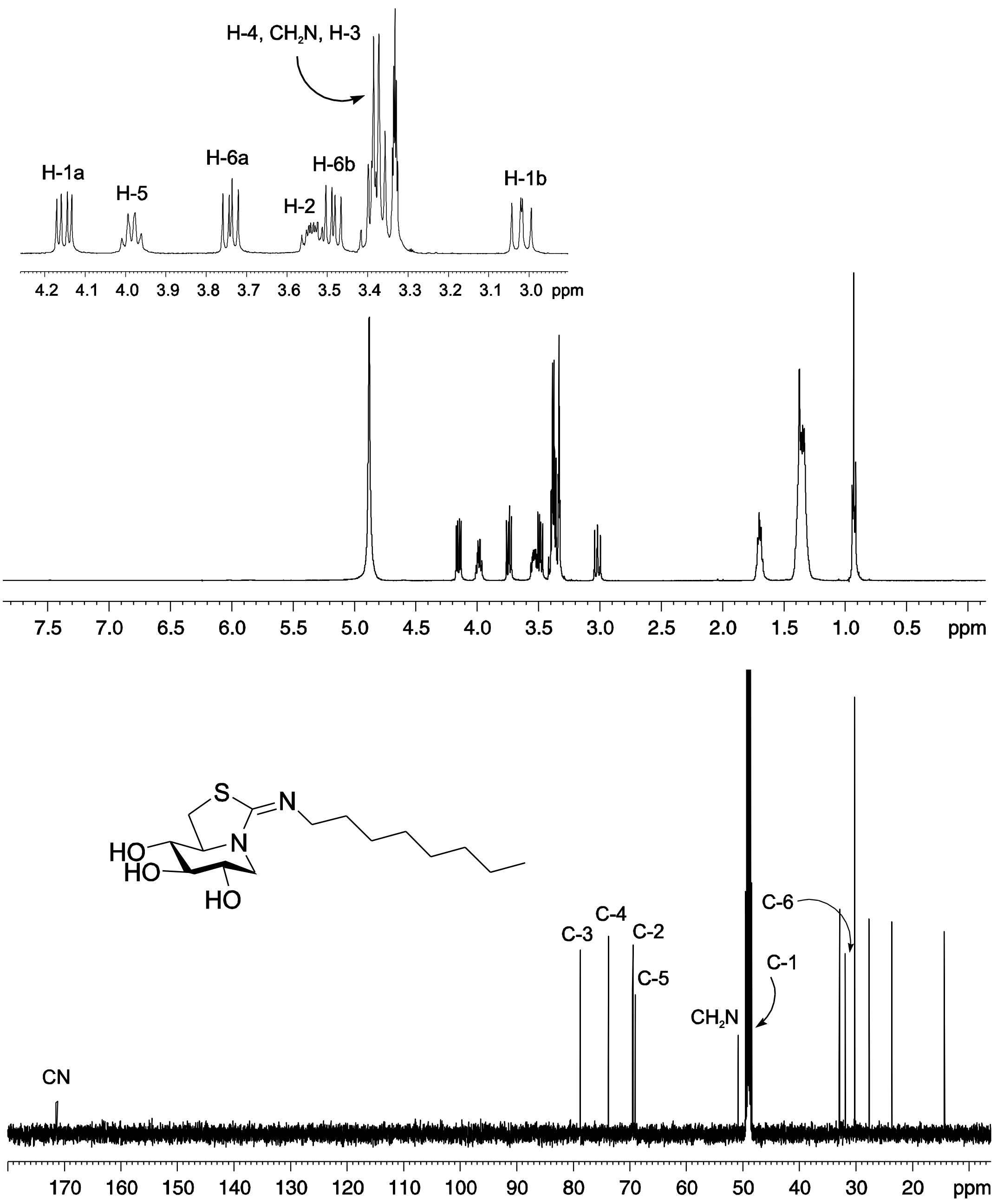

Figure S1. ^{1}H and ^{13}C RMN spectra (500 MHz, 125.7 MHz, CD_3OD) of **4**.

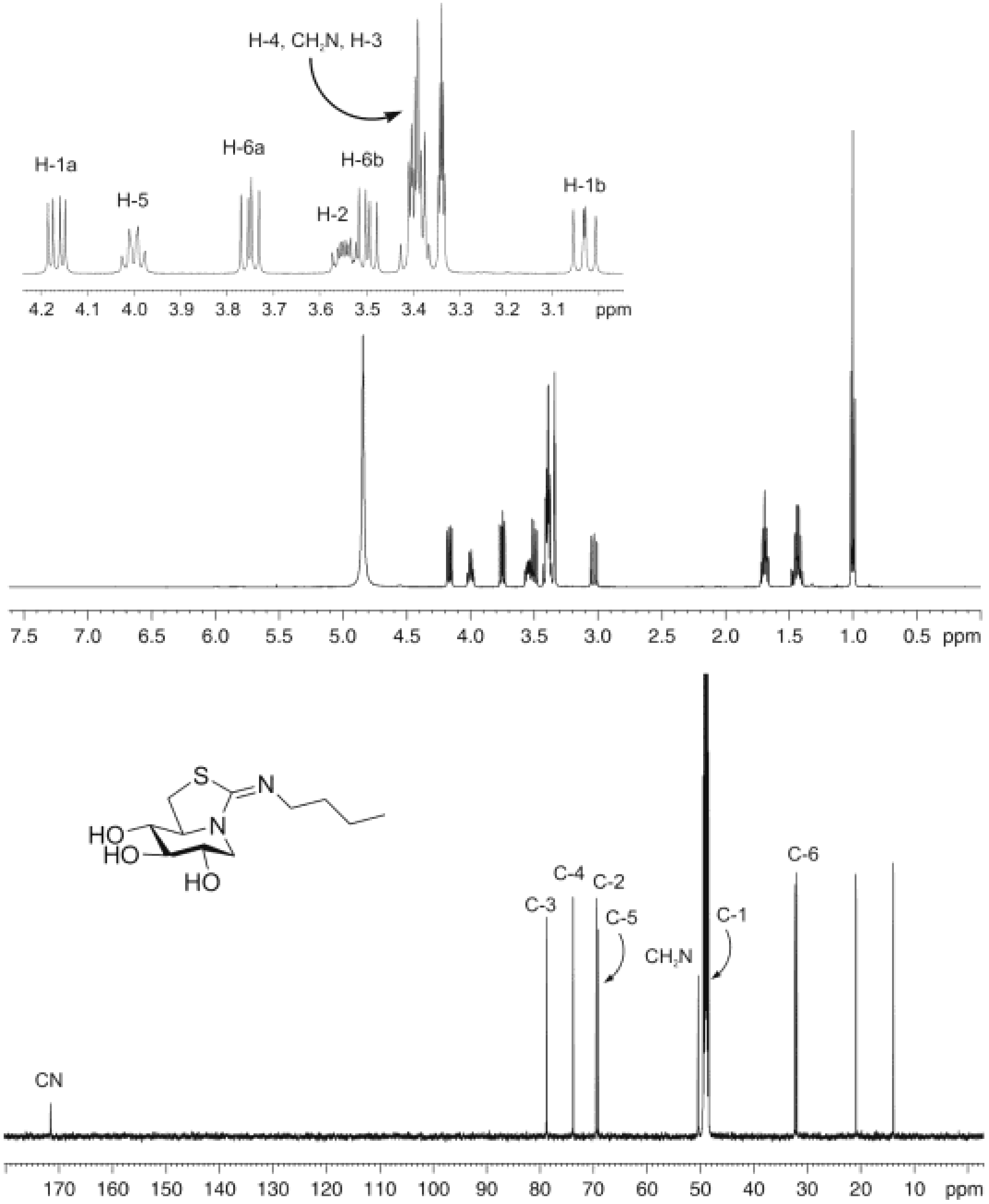

Figure S2. ^{1}H and ^{13}C NMR spectra (500 MHz, 125.7 MHz, CD_3OD) of **5**.

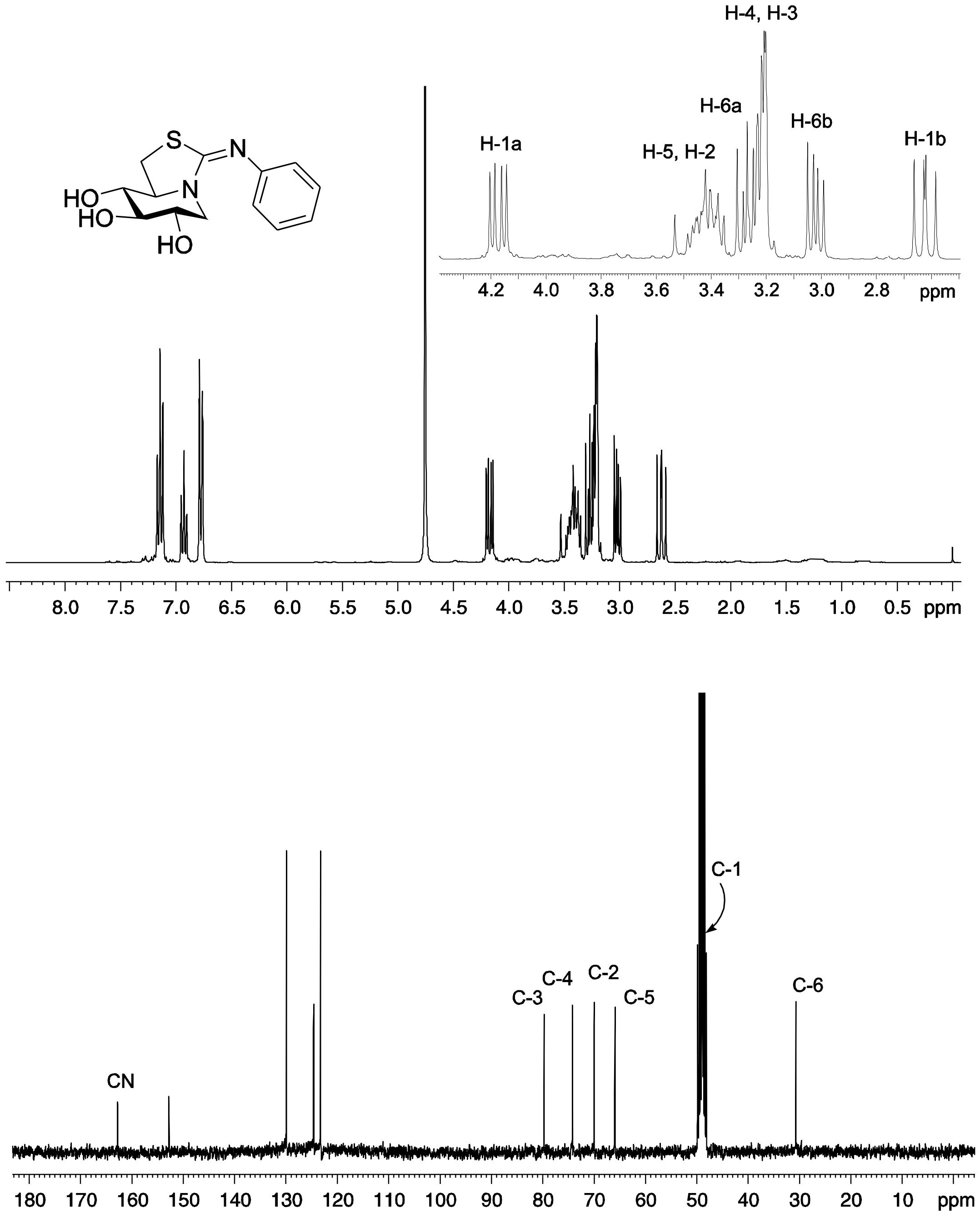

Figure S3. ^{1}H and ^{13}C NMR spectra (300 MHz, 75.5 MHz, CD_3OD) of **6**.

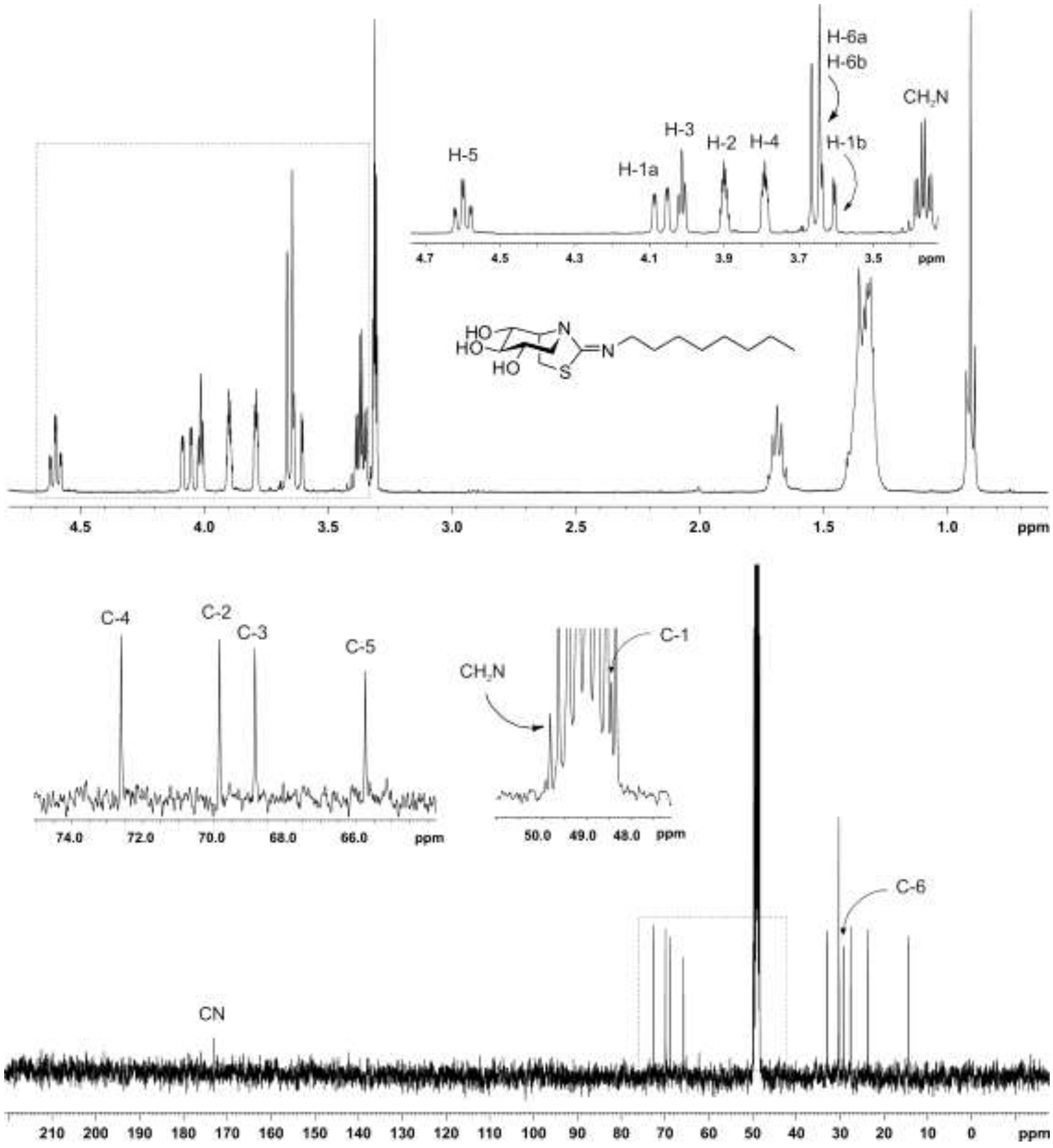

Figure S4. ^{1}H and ^{13}C NMR spectra (400 MHz, 100.6 MHz, CD_3OD) of **7**.

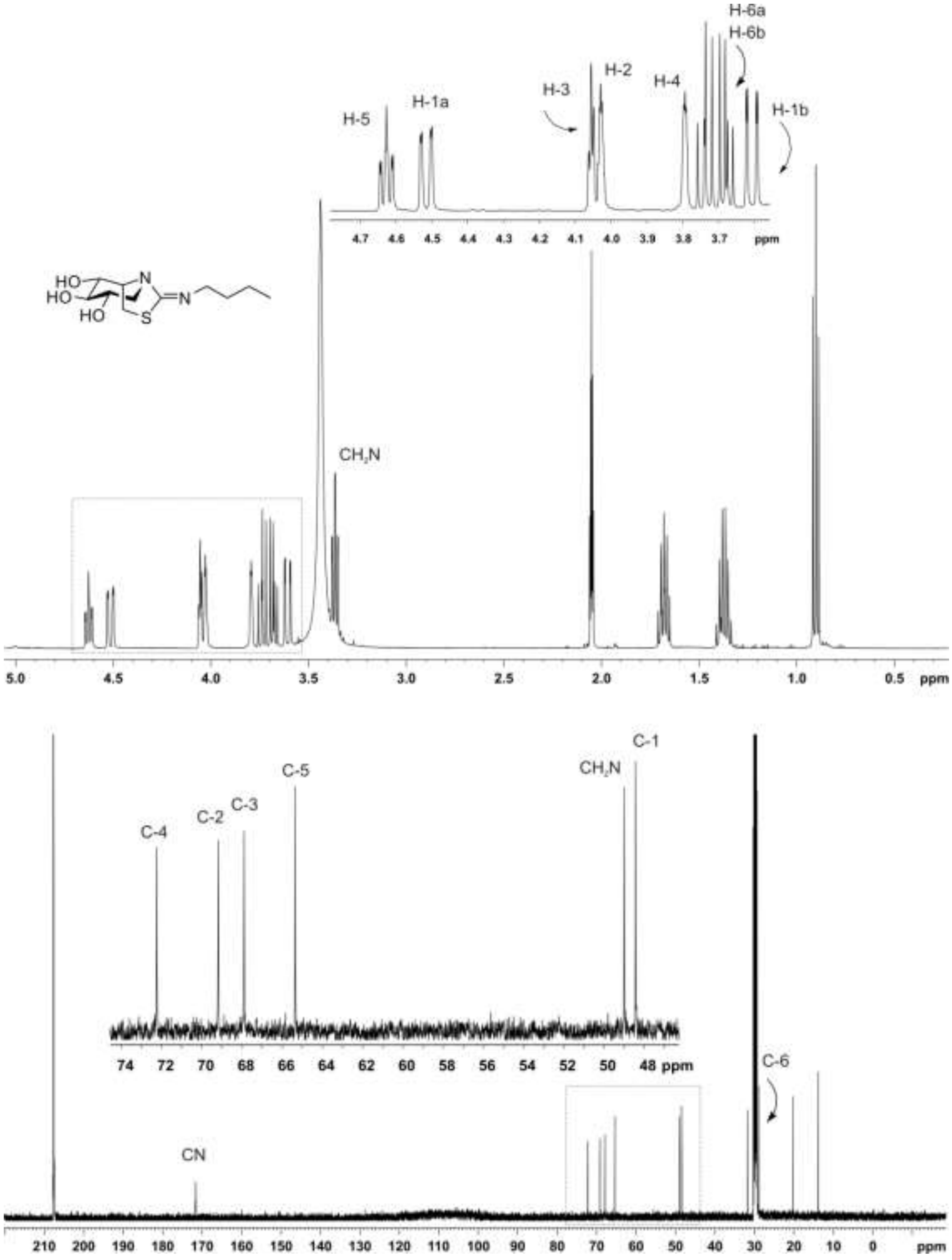

Figure S5. ^{1}H and ^{13}C NMR spectra (500 MHz, 125.7 MHz, 9:1 acetone-d_6-D_2O) of **8**.

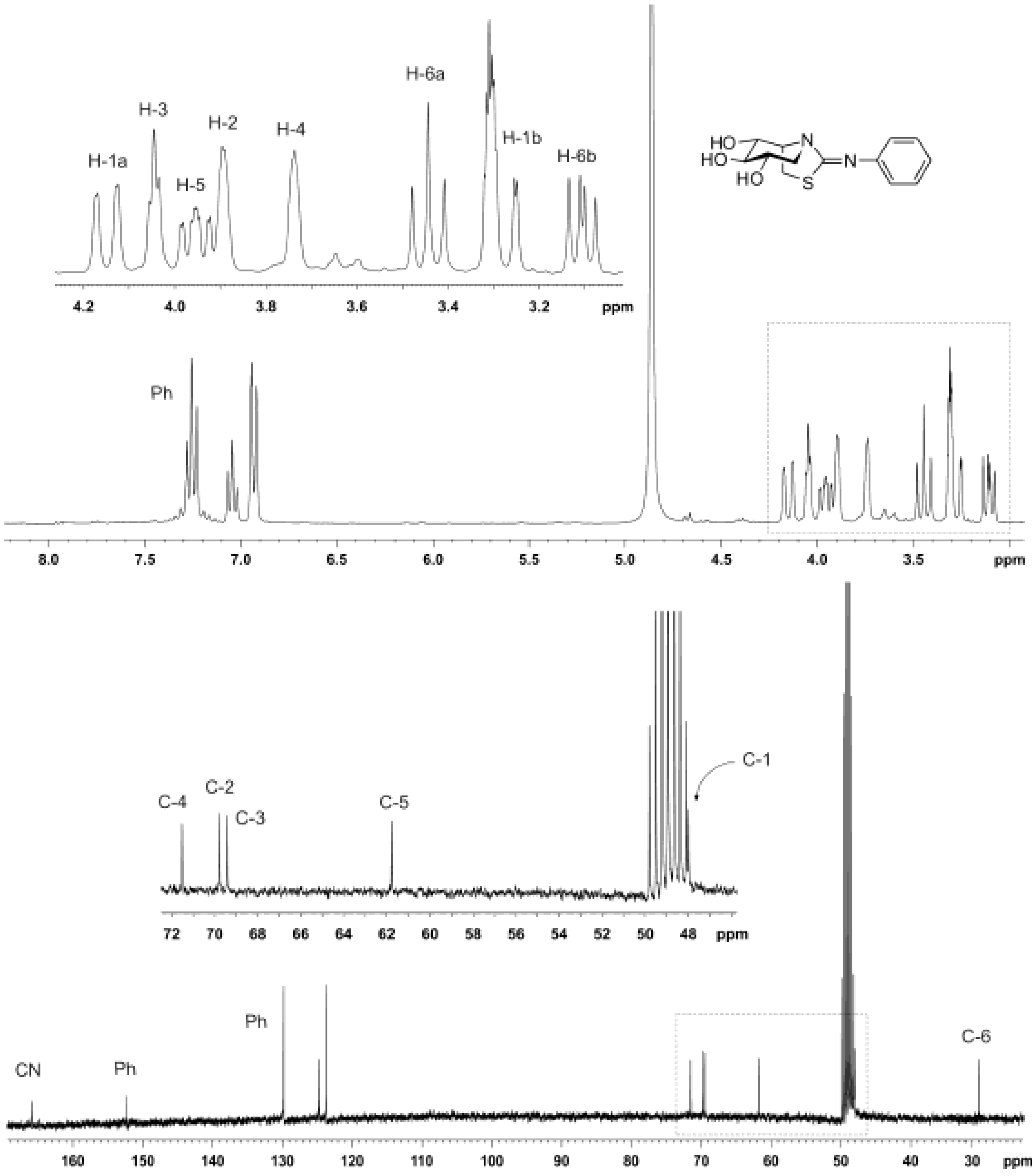

Figure S6. ^{1}H and ^{13}C NMR spectra (300 MHz, 75.5 MHz, CD_3OD) of **9**.

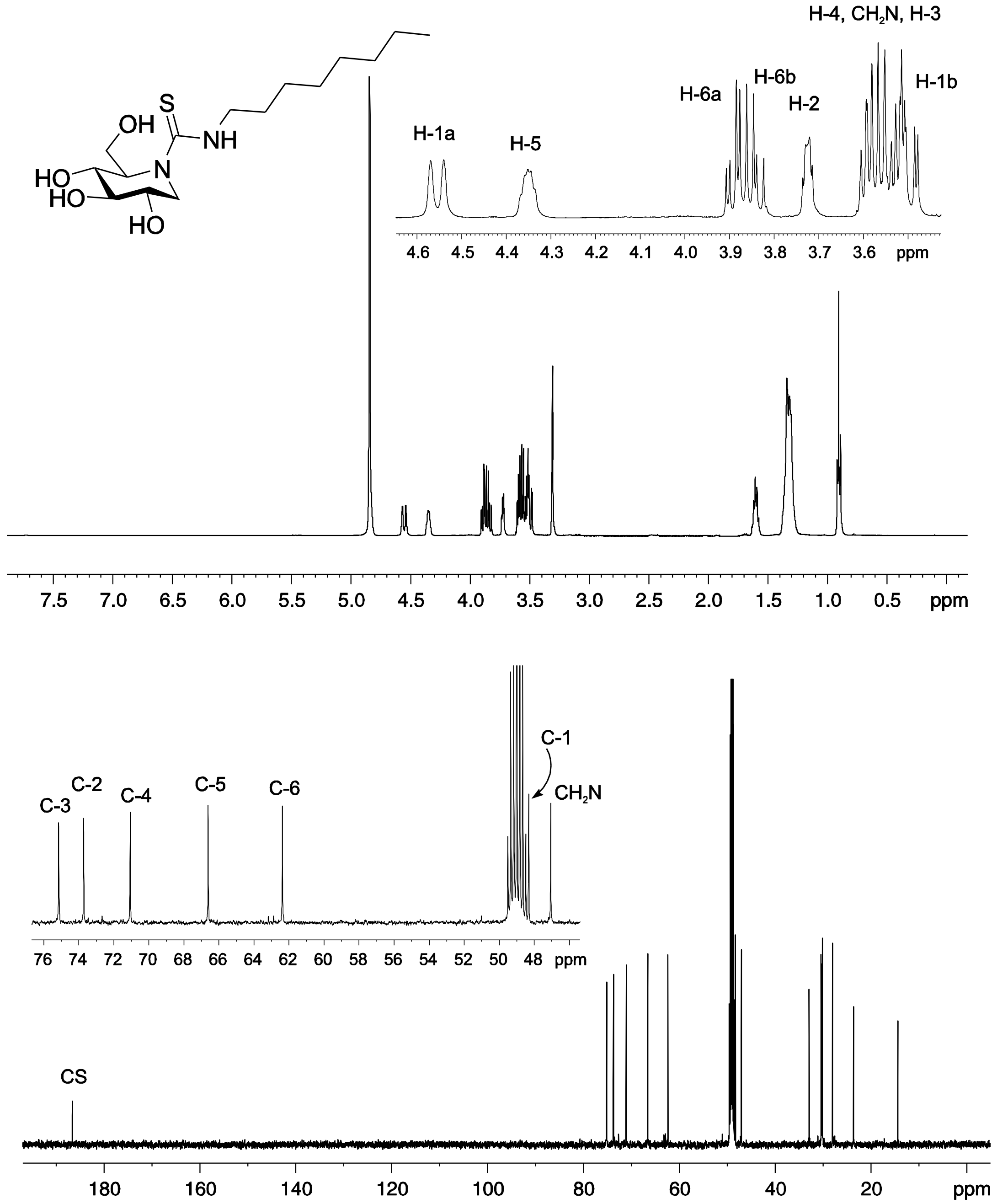

Figure S7. ^{1}H and ^{13}C RMN spectra (500 MHz, 125.7 MHz, CD_3OD) of **10**.

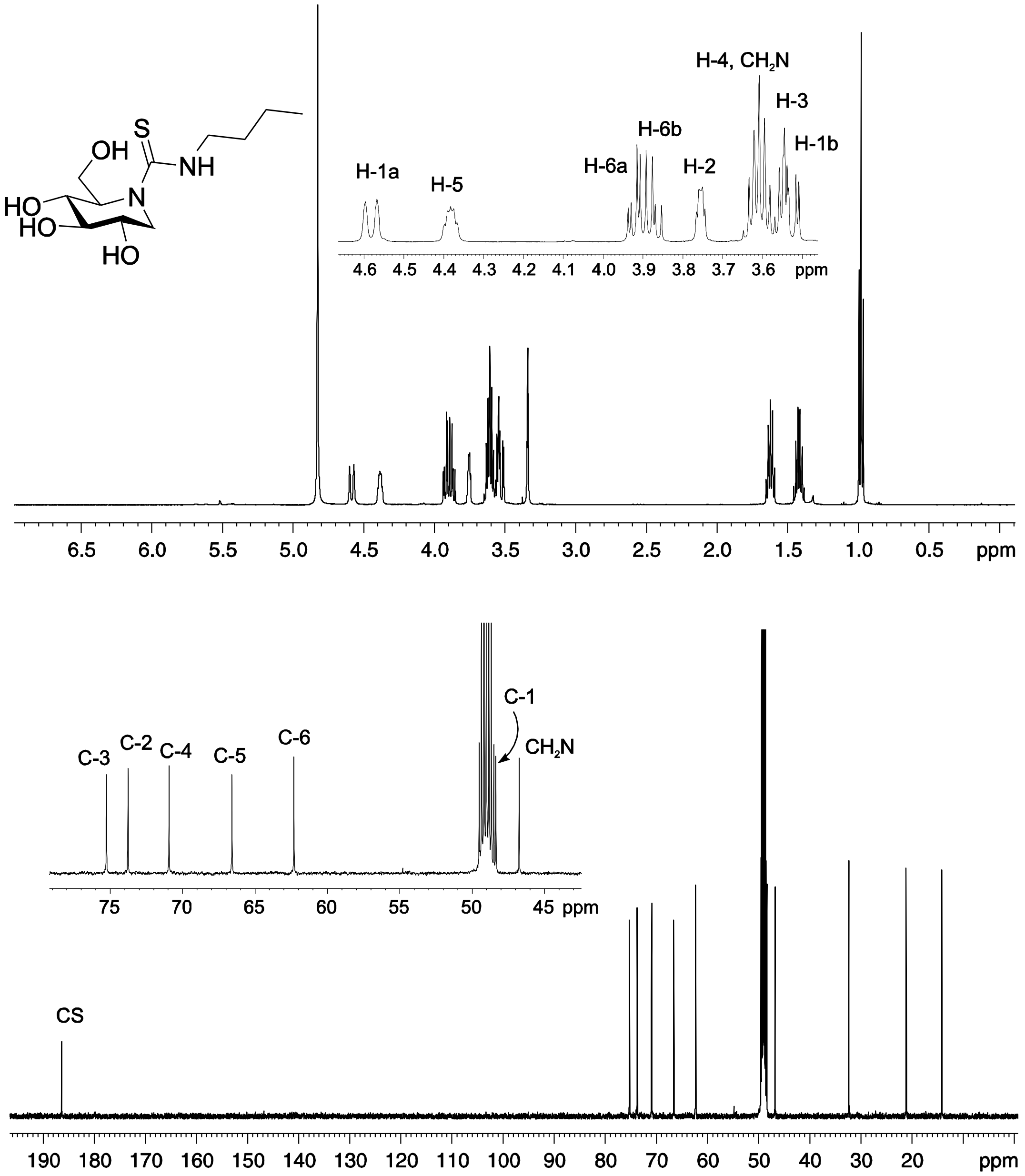

Figure S8. ^{1}H and ^{13}C NMR spectra (500 MHz, 125.7 MHz, CD_3OD) of **11**.

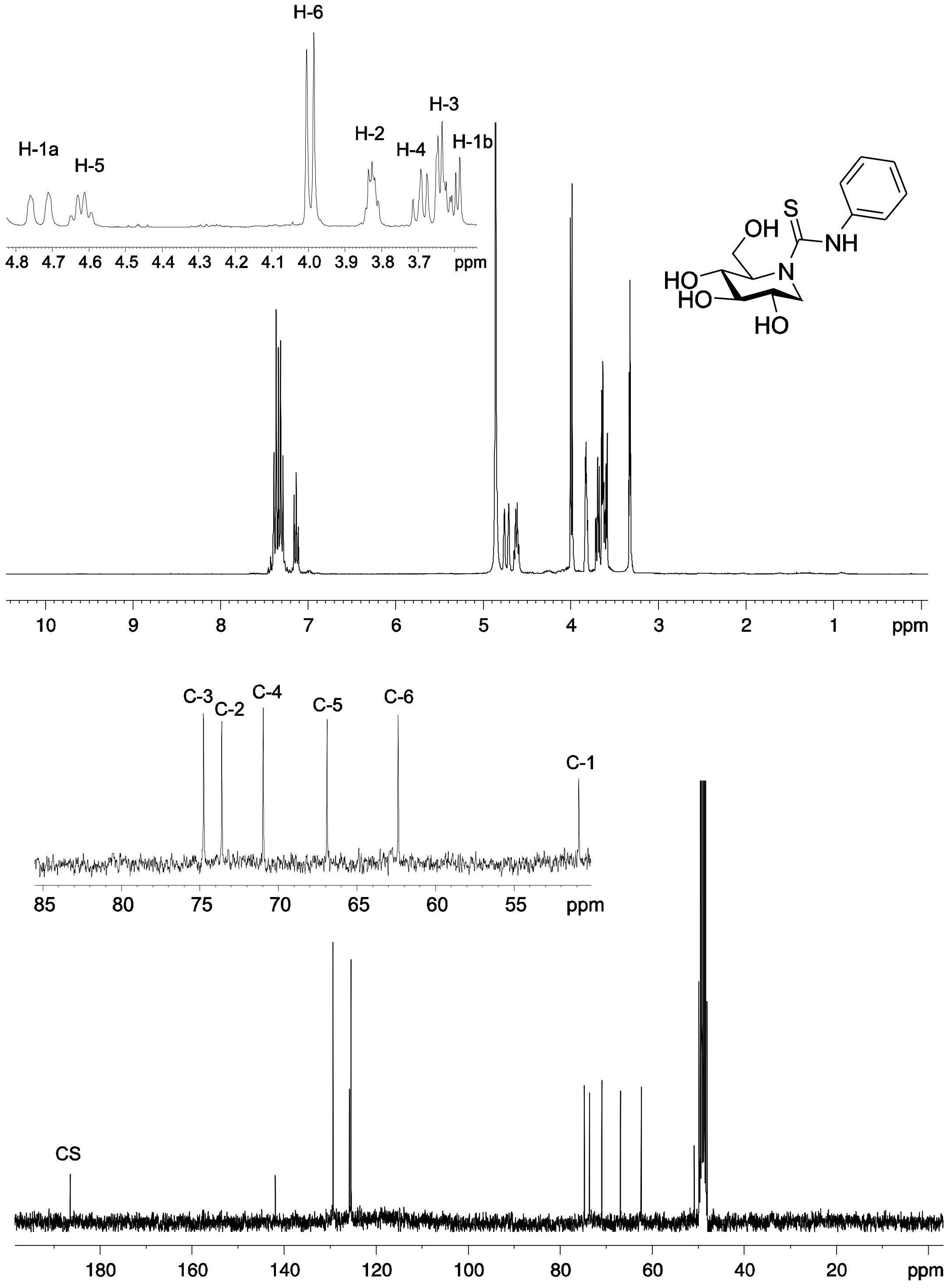

Figure S9. ^{1}H and ^{13}C NMR spectra (300 MHz, 75.5 MHz, CD_3OD) of **12**.

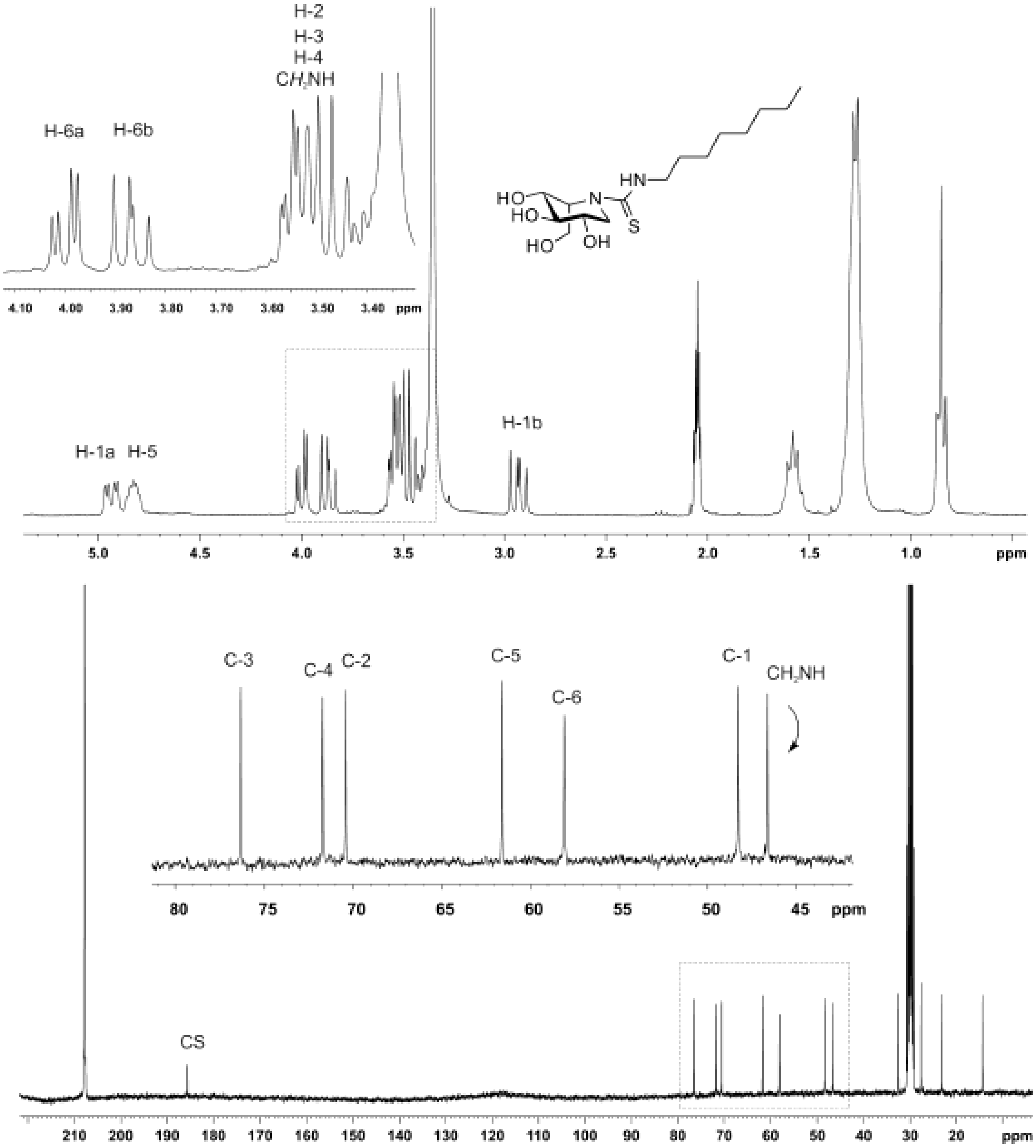

Figure S10. ^{1}H and ^{13}C NMR spectra (300 MHz, 75.5 MHz, 9:1 acetone-d_6-D_2O, 313 K) of **13**.

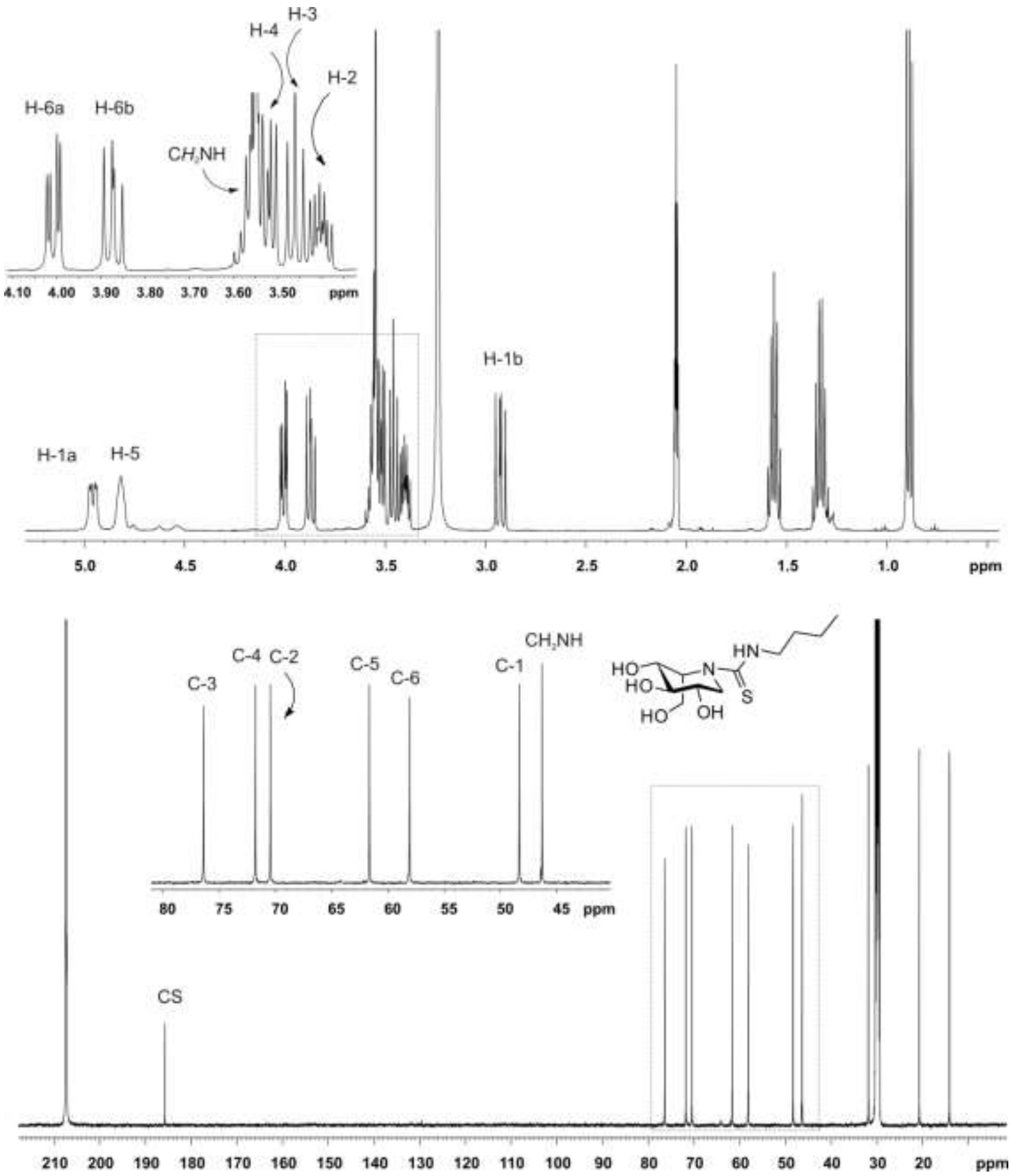

Figure S11. ^{1}H and ^{13}C NMR spectra (500 MHz, 125.7 MHz, 9:1 acetone-d_6-D_2O, 313 K) of **14**.

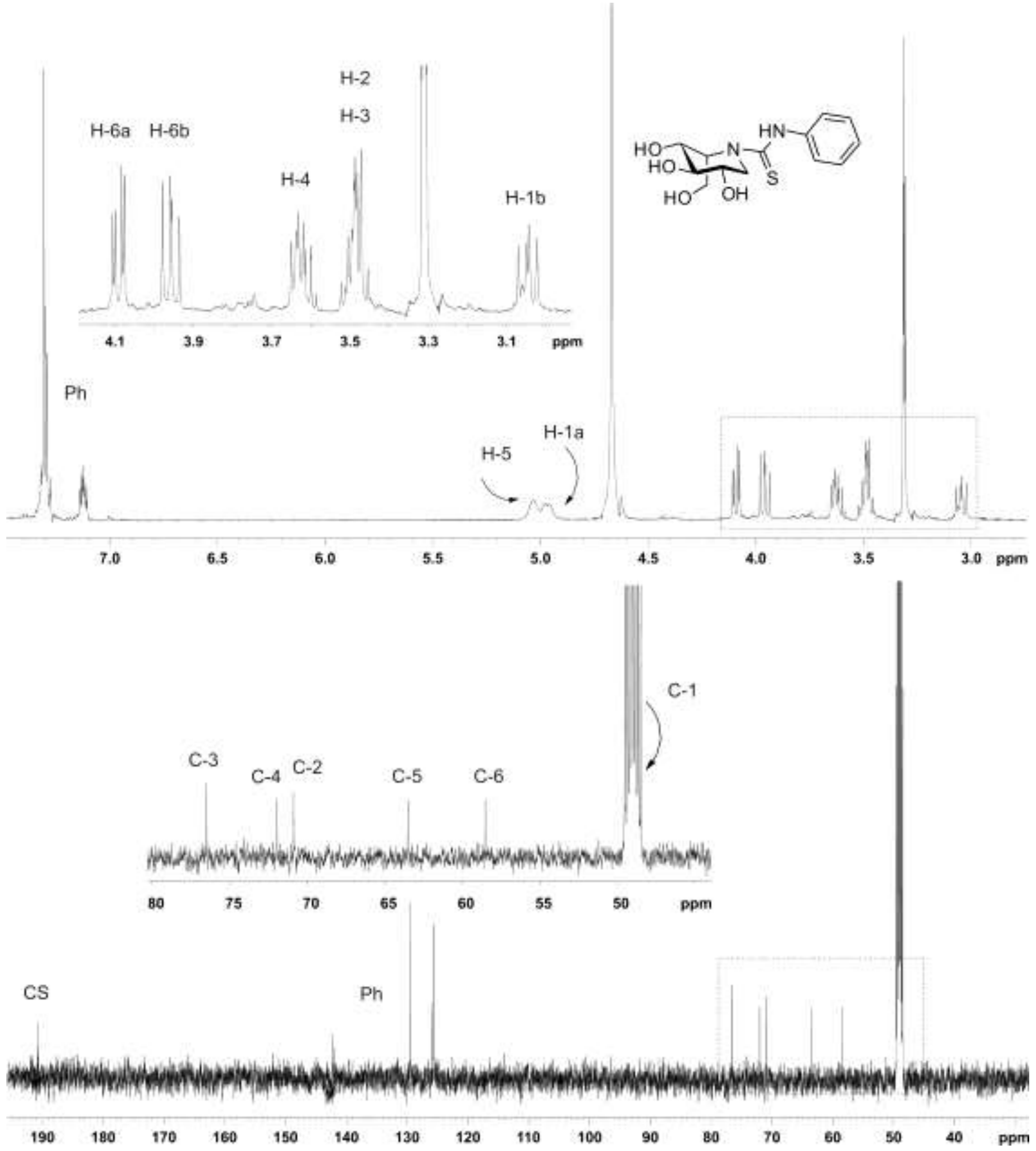

Figure S12. ^{1}H and ^{13}C NMR spectra (500 MHz, 125.7 MHz, CD_3OD, 313 K) of **15**.

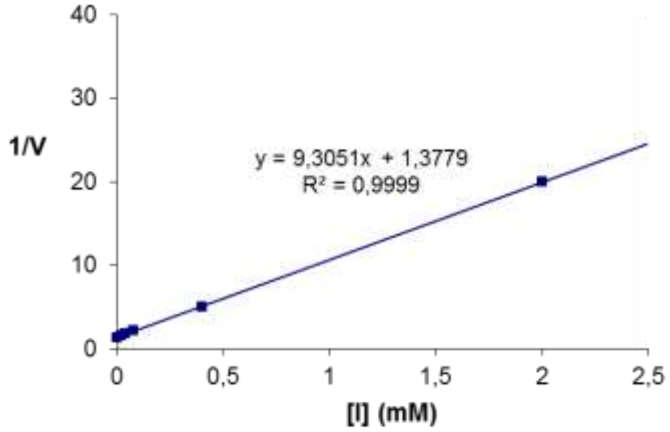

Figure S13. Dixon Plot for K_i determination (60±4 μM) of **12** against yeast maltase.

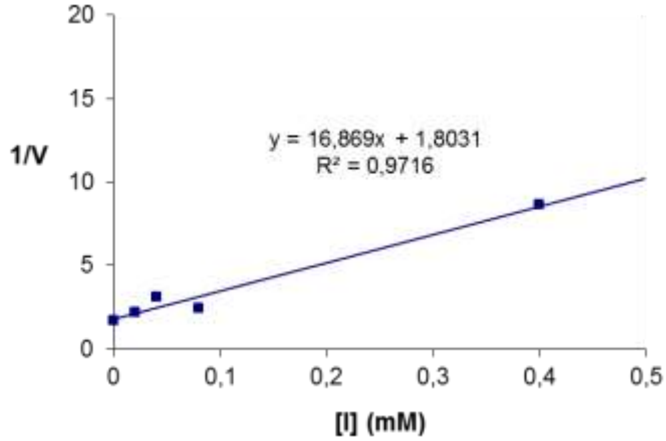

Figure S14. Dixon Plot for K_i determination (44±3 μM) of **6** against isomaltase.

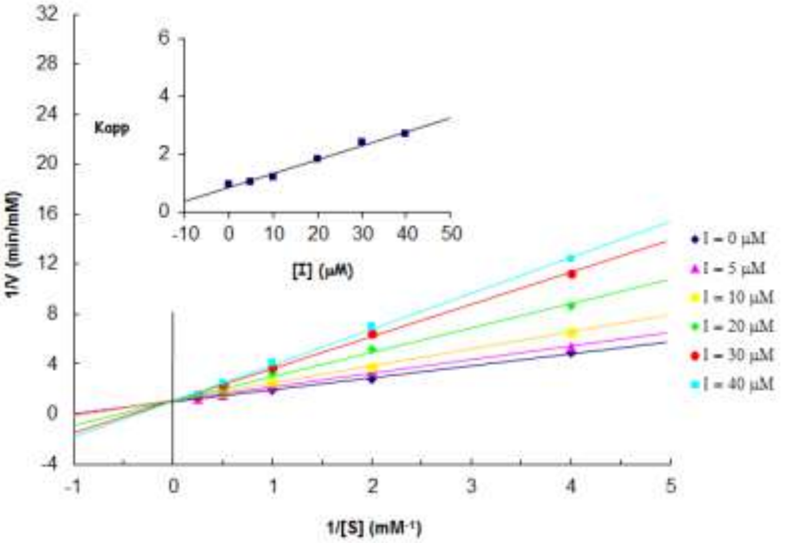

Figure S15. Lineweaver-Burk Plot for K_i determination (18± 2μM) of **12** against isomaltase.

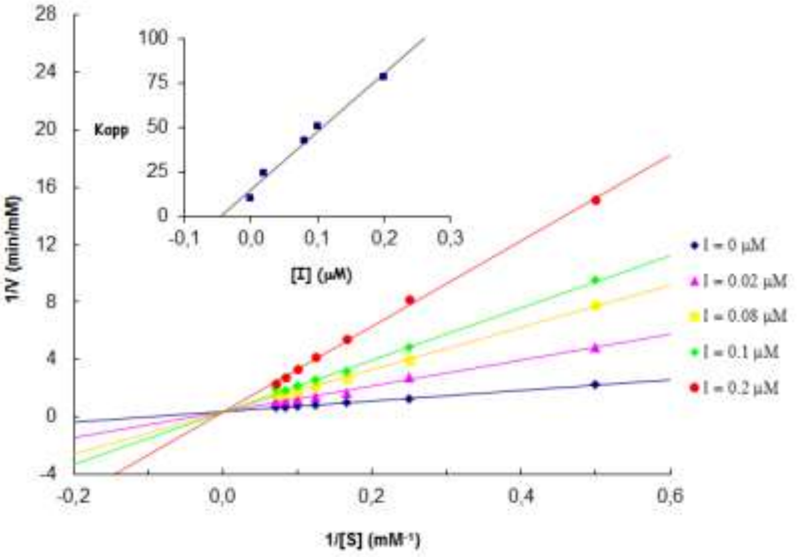

Figure S16. Lineweaver-Burk Plot for K_i determination (0.045±0.002 μM) of **4** against almonds β-glucosidase.

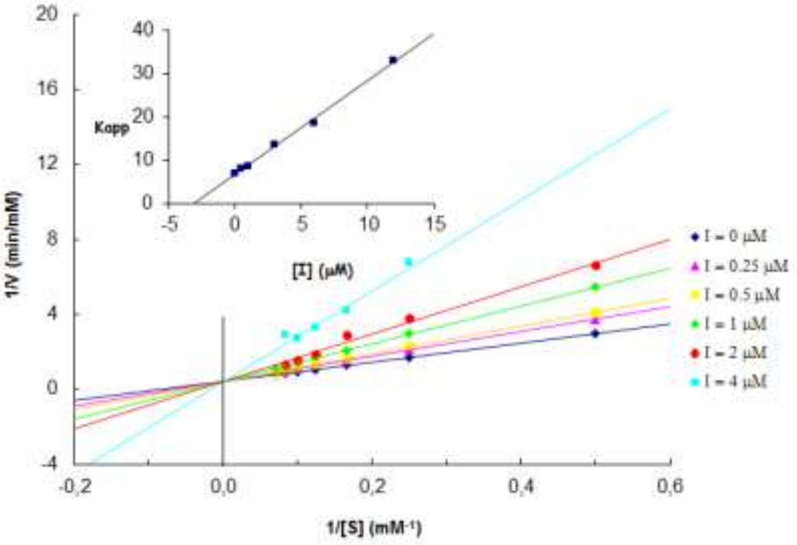

Figure S17. Lineweaver-Burk Plot for K_i determination (1.1±0.1 μM) of **5** against almonds β-glucosidase.

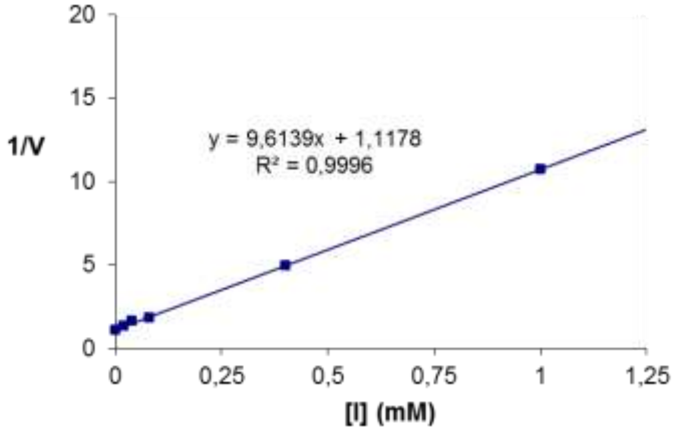

Figure S18. Dixon Plot for K_i determination (48±4 μM) of **6** against almonds β-glucosidase.

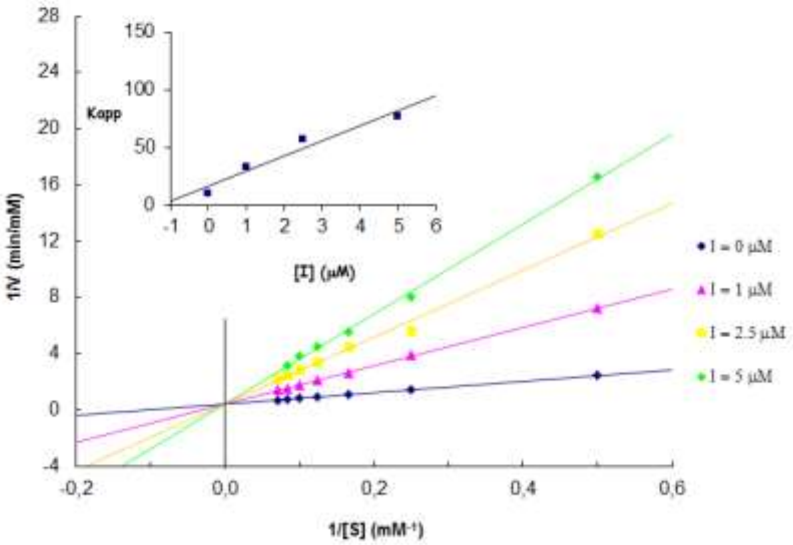

FigureS19. Lineweaver-Burk Plot for K_i determination (1.3±0.1 μM) of **10** against almonds β-glucosidase.

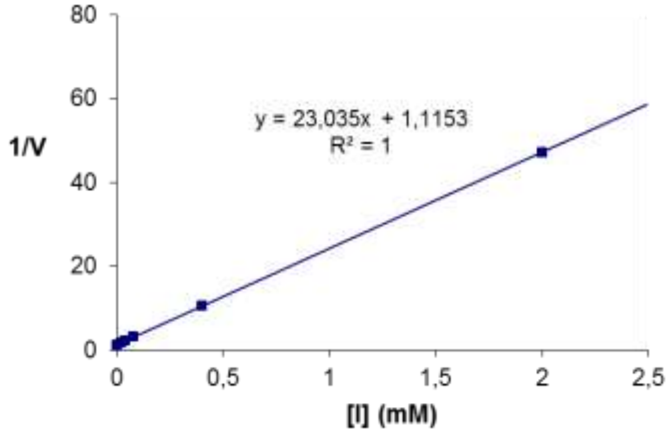

Figure S20. Dixon Plot for K_i determination (20±2 μM) of **11** against almonds β-glucosidase.

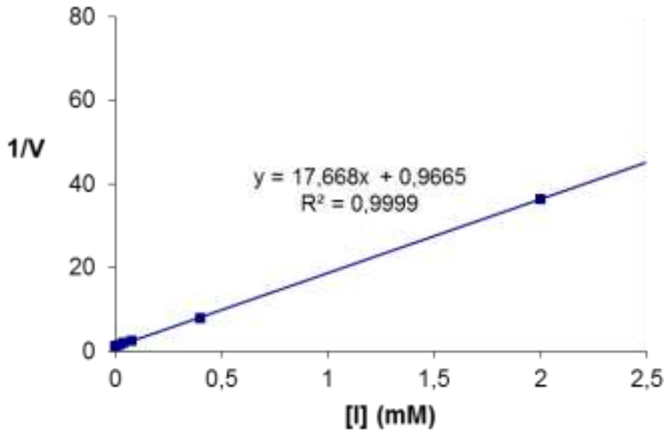

Figure S21. Dixon Plot for K_i determination (23±3 μM) of **12** against almonds β-glucosidase.

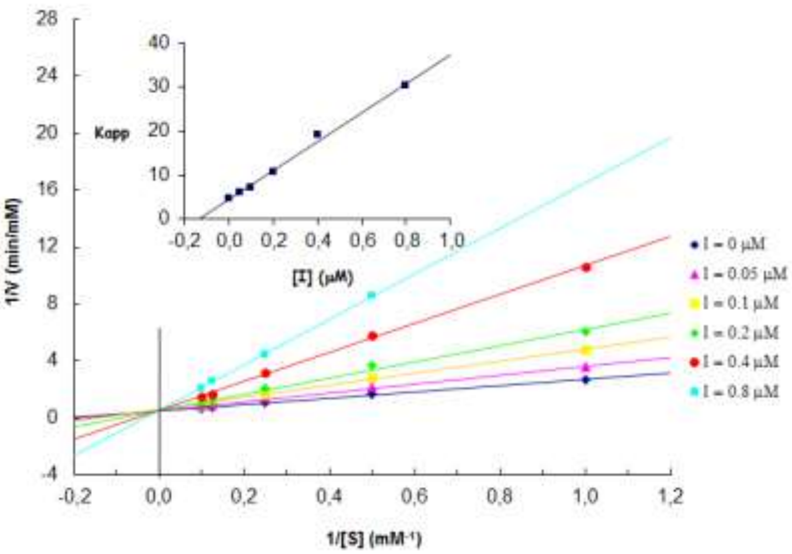

Figure S22 Lineweaver-Burk Plot for K_i determination (0.1±0.02 μM) of **4** against bovine liver β-glucosidase.

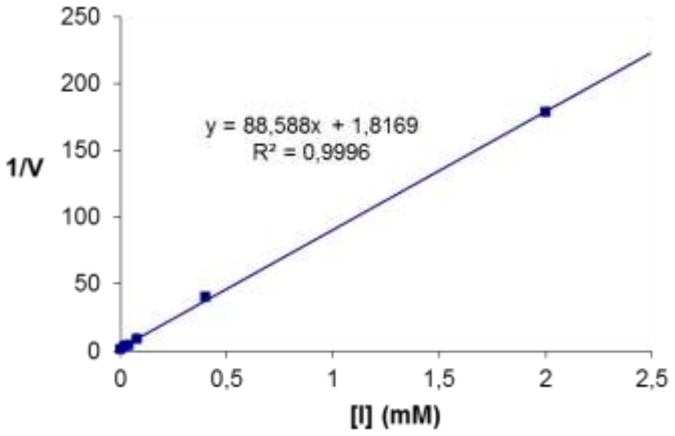

Figure S23. Dixon Plot for K_i determination (5.8±0.5 μM) of **5** against bovine liver β-glucosidase.

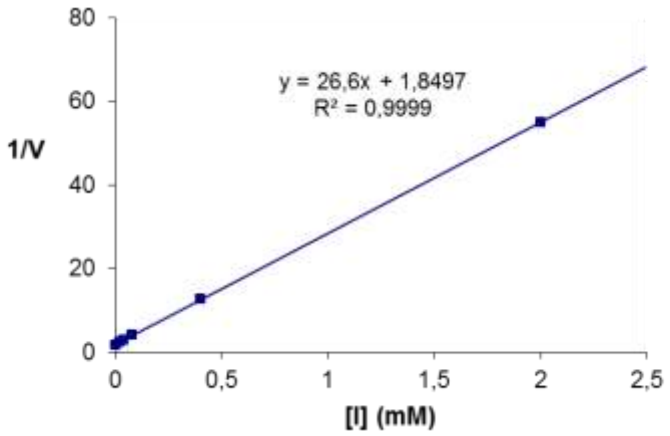

Figure S24. Dixon Plot for K_i determination (15±1 μM) of **6** against bovine liver β-glucosidase.

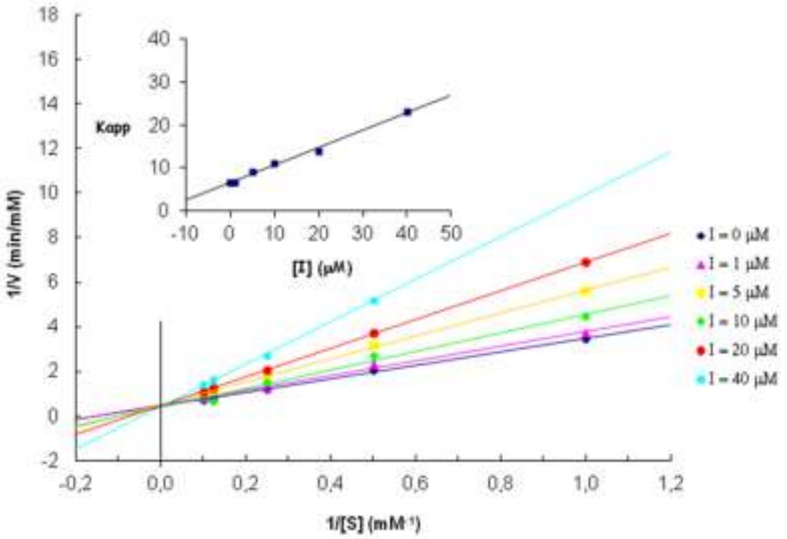

Figure S25. Dixon Plot for K_i determination (15±2 μM) of **7** against bovine liver β-glucosidase.

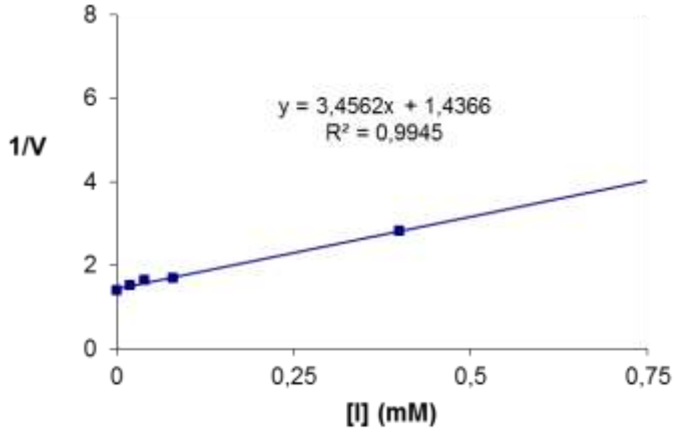

Figure S26. Dixon Plot for K_i determination (185±14 μM) of **8** against bovine liver β-glucosidase.

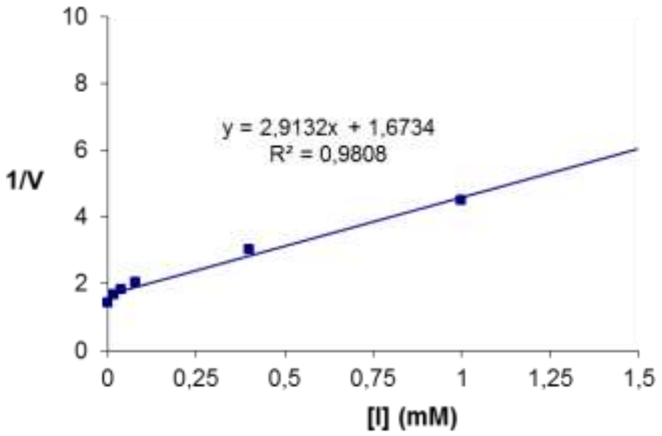

Figure S27. Dixon Plot for K_i determination (255±20 μM) of **9** against bovine liver β-glucosidase.

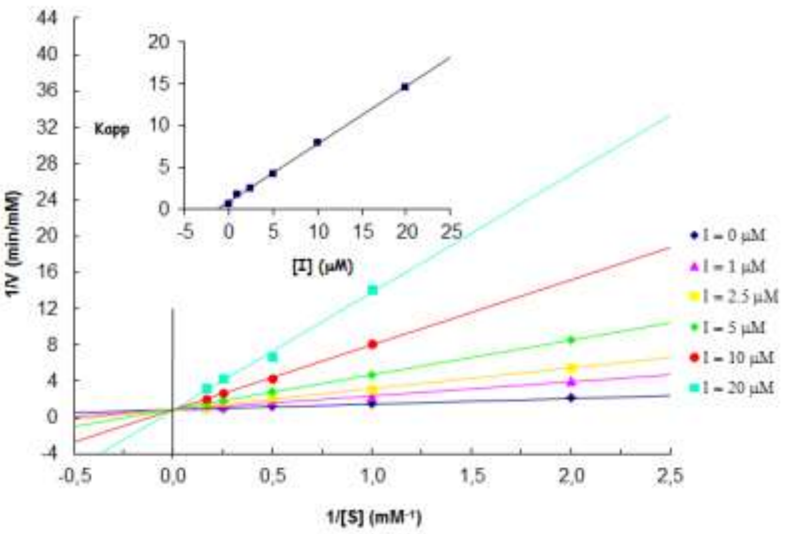

Figure S28. Lineweaver-Burk Plot for K_i determination (1.3±0.1 μM) of **10** against bovine liver β-glucosidase.

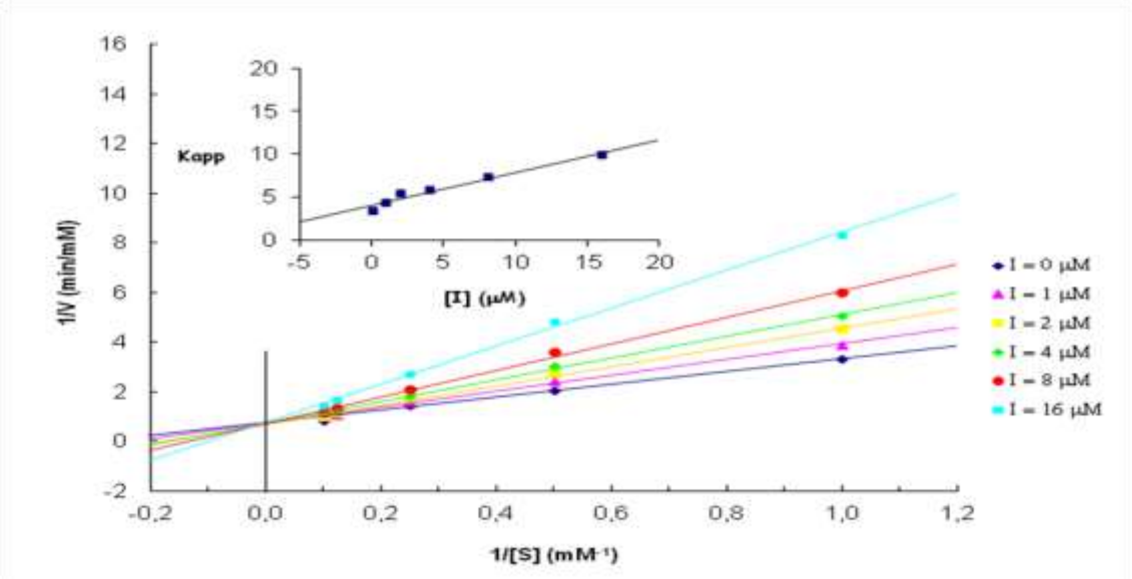

Figure S29. Lineweaver-Burk Plot for K_i determination (12.7±0.1 µM) of **11** against bovine liver β-glucosidase.

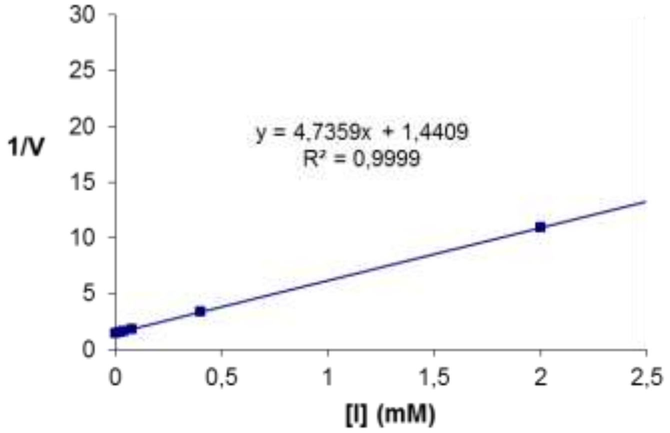

Figure S30. Dixon Plot for K_i determination (71±8 µM) of **12** against bovine liver β-glucosidase.

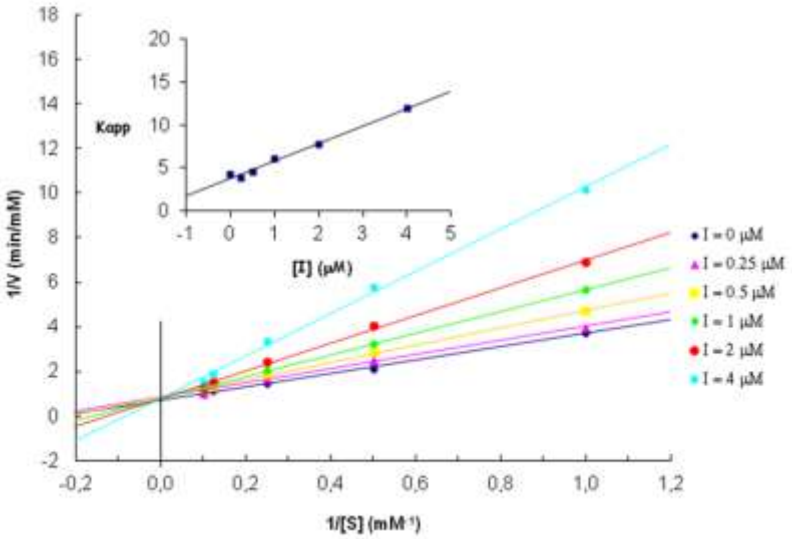

Figure S31. Lineweaver-Burk Plot for K_i determination (2.3±0.2 μM) of **13** against bovine liver β-glucosidase.

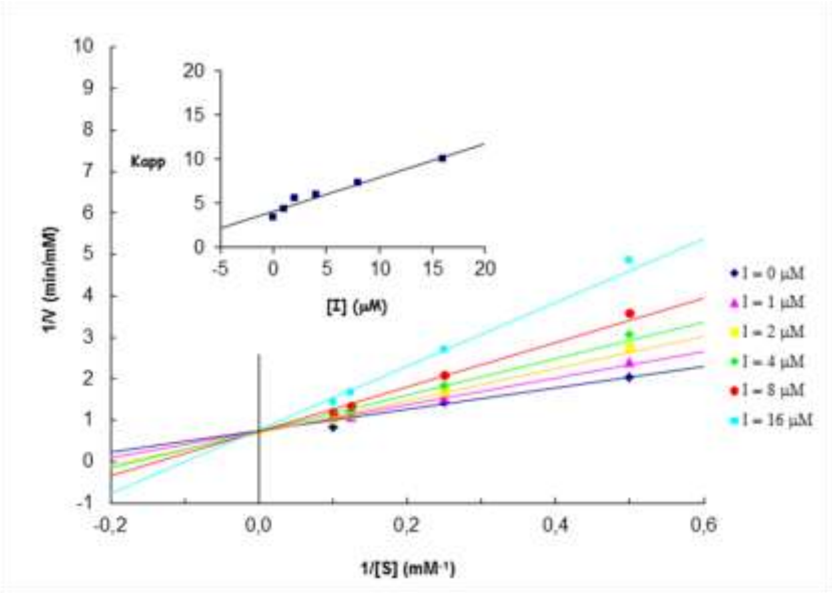

Figure S32. Lineweaver-Burk Plot for K_i determination (11±1 μM) of **14** against bovine liver β-glucosidase.

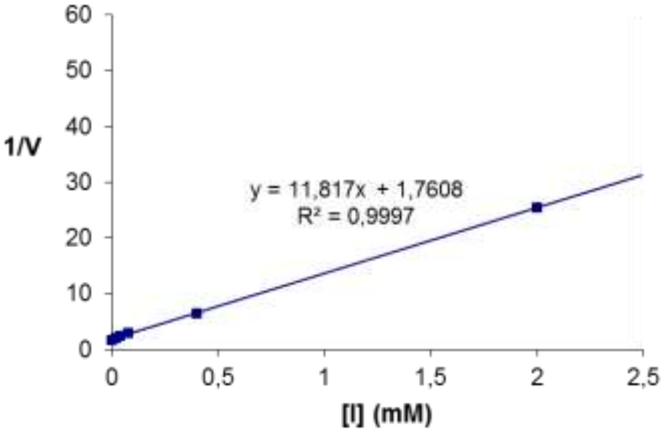

Figure S33. Dixon Plot for K_i determination (66±15 μM) of **15** against bovine liver β-glucosidase.
